# State of the Art in Sub-Phenotyping Midbrain Dopamine Neurons

**DOI:** 10.3390/biology13090690

**Published:** 2024-09-03

**Authors:** Valentina Basso, Máté D. Döbrössy, Lachlan H. Thompson, Deniz Kirik, Heidi R. Fuller, Monte A. Gates

**Affiliations:** 1 School of Medicine, Keele University, Staffordshire ST5 5BG, UK; v.basso@keele.ac.uk; 2 Laboratory of Stereotaxy and Interventional Neurosciences, Department of Stereotactic and Functional, Neurosurgery, Medical Center, University of Freiburg, 79106 Freiburg im Breisgau, Germany; mate.dobrossy@uniklinik-freiburg.de; 3 Department of Stereotactic and Functional Neurosurgery, Medical Center, University of Freiburg, 79106 Freiburg im Breisgau, Germany; 4 Faculty of Biology, University of Freiburg, 79104 Freiburg im Breisgau, Germany; 5 Charles Perkins Centre, Faculty of Medicine and Health, School of Medical Sciences, The University of Sydney, Sydney, NSW 2006, Australia; lachlan.thompson@sydney.edu.au; 6 Aligning Science Across Parkinson’s (ASAP) Collaborative Research Network, Chevy Chase, MD 20815, USA; 7 Brain Repair and Imaging in Neural Systems (B.R.A.I.N.S) Unit, Department of Experimental Medical Science, Lund University, BMC D11, 22184 Lund, Sweden; deniz.kirik@med.lu.se; 8 School of Pharmacy and Bioengineering, Keele University, Staffordshire ST5 5BG, UK; 9 Wolfson Centre for Inherited Neuromuscular Disease, TORCH Building, RJAH Orthopaedic Hospital, Oswestry SY10 7AG, UK

**Keywords:** substantia nigra pars compacta (SNpc), ventral tegmental area (VTA), dopamine (DA), midbrain dopaminergic neurons (mDA), Parkinson’s disease (PD), schizophrenia (SZ), major depression, drug addiction

## Abstract

**Simple Summary:**

This review delves into the differences between two important groups of dopamine-producing brain cells, found in regions called the ventral tegmental area and the substantia nigra. While these cells are located close to each other, they play different roles in the brain and are affected differently by diseases like Parkinson’s. Understanding these differences is key to developing better treatments and more selective, without causing unwanted effects on other brain functions. This review discusses how these cells are structured, how they connect to other parts of the brain, how they function, and the differences during development. The goal is to provide insights that could lead to more precise therapies, targeting specific groups of cells without harming others. This research is important for advancing treatments for brain disorders and improving our understanding of the brain’s complex wiring.

**Abstract:**

Dopaminergic neurons in the ventral tegmental area (VTA) and the substantia nigra pars compacta (SNpc) comprise around 75% of all dopaminergic neurons in the human brain. While both groups of dopaminergic neurons are in close proximity in the midbrain and partially overlap, development, function, and impairments in these two classes of neurons are highly diverse. The molecular and cellular mechanisms underlying these differences are not yet fully understood, but research over the past decade has highlighted the need to differentiate between these two classes of dopaminergic neurons during their development and in the mature brain. This differentiation is crucial not only for understanding fundamental circuitry formation in the brain but also for developing therapies targeted to specific dopaminergic neuron classes without affecting others. In this review, we summarize the state of the art in our understanding of the differences between the dopaminergic neurons of the VTA and the SNpc, such as anatomy, structure, morphology, output and input, electrophysiology, development, and disorders, and discuss the current technologies and methods available for studying these two classes of dopaminergic neurons, highlighting their advantages, limitations, and the necessary improvements required to achieve more-precise therapeutic interventions.

## 1. Introduction

Dopamine (DA), also known as 4-(2-aminoethyl)-1,2-benzenediol, was identified as an independent neuromodulator in 1957 [1]. The synthesis of dopamine starts from L-tyrosine, which is converted by tyrosine hydroxylase (TH) to L-DOPA and subsequently to dopamine by the aromatic enzyme-L-amino-acid decarboxylase (AADC or DDC) [2,3,4,5]. After its synthesis, dopamine is stored in presynaptic vesicles via vesicular monoamine transporter 2 (VMAT2) until there is a stimulus that induces its release [6]. Released dopamine can be re-uptake by the dopamine transporter (DAT) [7].

Carlsson, Falck, and Hillarp, using the formaldehyde histofluorescence method, were the first to visualize two groups of catecholamines (CAs) in the mammalian brain: noradrenaline (NA) and dopamine (DA) [8]. Only two years later, twelve groups of CA cells classified between “Area 1” (A1) to A12 were identified, and subsequently, five other groups were added to this classification (A13–A17) [9]. The first twelve are distributed from the medulla oblongata to the hypothalamus, while the other five are localized in the diencephalon, olfactory bulb, and retina [10]. Currently, the most commonly used method to visualize dopaminergic neurons is staining for tyrosine hydroxylase (TH) [11]. Overall, this classification of CAs, even though it was made 70 years ago, is still useful for comparing different vertebrates because it provides a standardized framework for studying and understanding the similarities and differences in catecholamine systems across species. For a graphic representation of catecholaminergic neurons and their major projections in the rat brain, refer to Figure 7 of [12]. For localization and axonal projection specifically of dopaminergic cell groups in the mammalian adult brain, refer to Figure 1 of [13].

**Figure 1:**
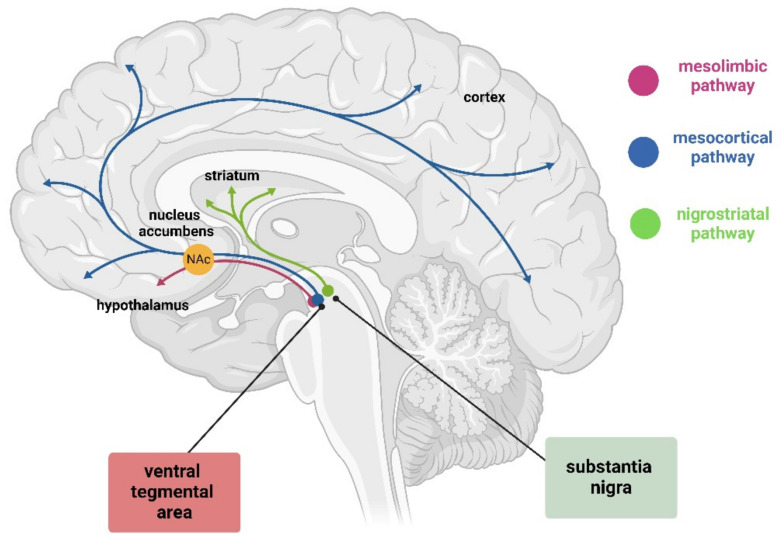
Graphic representation of dopamine pathway in the brain. This pathway includes the mesolimbic pathway (pink) from the VTA to nucleus accumbens (orange, the mesocortical pathway (blue) from the VTA to cortex, and the nigrostriatal pathway (green) from SN to striatum. Created with BioRender (https://www.biorender.com/).

The A9 region refers to the substantia nigra pars compacta (SNpc), whereas A10 indicates the ventral tegmental area (VTA) (Figure 1). Together with the retrorubral field (A8), they contain almost 75% of the dopamine neurons in the brain [14]. A9 has been associated with locomotion/motor functions, while A10 has been associated with reward-orientated behaviors and motivation. The progressive loss of dopaminergic neurons in the SNpc is associated with the insurgence of Parkinsons’s disease (PD) [15,16]. On the other hand, the VTA malfunction and pathology is correlated with schizophrenia, and new evidence links this area also to depression, drug addiction, and other psychiatric disorders [17,18,19]. While these two areas show diversification, they also share many commonalities, making it challenging to understand how a single neurotransmitter system could be responsible for such disparate symptoms and apparently unrelated diseases. Many hypotheses and explanations have been proposed over the years, but none completely explains this phenomenon.

This review will summarize our current understanding of SNpc and VTA dopaminergic neurons, which could explain the diversity in their function despite being linked to one another during development and in adulthood through interconnected networks. Even though knowledge about the differences between the SNpc and VTA is increasing, we have not yet developed a way to differentiate the two groups selectively with reliable molecular biomarkers, nor are we able to target them pharmacologically. We discuss this lack of selectivity in the context of limitations for research purposes and the collateral effects associated with current treatments of PD and schizophrenia. In the latter part of this review, we present an overview of the methods and innovations that have advanced the study of these two areas and underline the limitations and advantages of the techniques and additional needs to enable a deeper understanding of the significance of these discoveries.

## 2. Similarities and Differences between the VTA and SNpc

### 2.1. Anatomical Localization and Structure

The substantia nigra (SN) is localized in the midbrain’s floor posterior to the crus cerebri fibers of the cerebral peduncle. Primates and rodents have two SNs, one on the right and one on the left hemisphere of the brain. Each SN is composed of two major parts: the pars reticulata (SNpr), with gamma-aminobutyric acid-containing (GABAergic) neurons; and the SNpc, which contains most of the dopaminergic neurons. In monkeys and humans, a further division is suggested, pars lateralis, which resembles for the most part the remainder of pars reticulata, except for the bigger dimension in the cell bodies [20,21]. The SNpc, in turn, has been characterized according to two tiers: a dorsal and ventral tier. The dorsal tier includes the dorsal SNpc and the continuous VTA. It forms a mediodorsal bond of cells between the SNpc and VTA. The ventral tier constitutes a densocellular region and the columns (also called ventral group) [22]. The SNpc in humans is easily identifiable visually due to its dark color, which is caused by its high neuromelanin content and gives it the Latin name that literally means “dark substance” [20].

The VTA is positioned medial to the SNpc juxtapositioned to the mid-sagittal line. It has a heterogeneous composition and lacks clear boundaries, making it initially difficult to separate from the SN. The VTA group is composed of several subregions: parabrachial pigmented nucleus (PBP), paranigral nucleus (PN), caudal linear nucleus (CLi), interfascicular nucleus (IF), and rostral linear nucleus of the raphe (RLi). The scientific community agrees that the PBP and PN are part of the VTA, but there is no consensus on whether the other three subpopulations are to be included or not as part of VTA [23]. Like the SN, the VTA contains different types of neurons, including dopaminergic, GABAergic, and glutamatergic neurons; moreover, neurons that co-release two of these are also reported, like dopamine and glutamate, making the studying of VTA even more complicated [23,24,25]. Due its complex structure and the lack of concordance in the regions that are part of VTA, it is important to compare information from different studies to indicate how the experiments were performed and which areas where considered.

In addition to their close localization and the fact that they contain most of the dopaminergic neurons, both the VTA and the SNpc are subdivided into distinct subdomains that are correlated with different characteristics, distinct functions and innervation targets. We will not delve deeply into this topic in this review, but for more information, please refer to Garritsen et al. (2023) [26]. The number of dopaminergic neurons distributed within the two areas is different. In humans, the number of dopaminergic neurons in the SNpc ranges between 200,000 and 420,000, while it ranges between 21,000 25,000 in rats and 8000 12,000 in mice. The number of dopaminergic neurons in the ventral tegmental area (VTA) ranges from 60,000 to 65,000, 20,000 to 40,000, and 8000 to 12,000 for humans, rats, and mice, respectively. When extrapolating observations made in rodents to humans, it is important to remember that the difference in the number of neurons between the SNpc and VTA in humans is much larger than the one in rodents [27].

### 2.2. Dopaminergic Neurons Output and Input

The dopaminergic neurons of the VTA send long projections to the limbic and cortical regions via the mesocortical and mesolimbic pathways, both of which travel through the medial forebrain bundle. The mesolimbic DA axons progress to the nucleus accumbens (NAc), which receives the densest innervation from the VTA, targeting both the core and shell subdomains. Some fibers remain in the NAc, while others continue to the terminal targets, such as the dorsal striatum and other limbic areas, including the amygdala and hippocampus. Meanwhile, the mesocortical DA fibers travel through the medial forebrain bundle to reach the prefrontal cortex (PFC) directly or by passing through the NAc and STR and external capsule (Figure 1) [28]. The mesocorticolimbic system participates in the control and regulation of a variety of functions, including fear, aversion, and reward; positive and negative reinforcement; motivated behavior; working-, place-, and reward-associated memory; object recognition; decision-making; cognitive and executive functions; goal-directed behaviors; temporal control; and food-intake [29,30,31,32,33,34,35,36,37,38]. It is important to mention that the mesocorticolimbic system is one of the last neuronal circuits in the brain to mature. Observations in rodents have shown that this circuitry is constantly changing from embryonic life through adolescence, achieving complete maturation during adolescence [11,39,40,41].

DA neurons of the SNpc project via the medial forebrain bundle, specifically via the nigrostriatal pathway to the dorsal striatum, with only a small number projecting to the ventral striatum and cortex (Figure 1) [42]. These neurons form synapses with two populations of neurons according to their receptor (D1-like and D2-like receptor family), which lead to movement activation or suppression. The striatal neurons expressing D1-like receptor family project to the globus pallidus internus (GPi) through the direct pathway, while neurons expressing D2-like receptor family project to the globus pallidus externus (GPe) through the indirect pathway [43]. The projections that reach the GPi send inhibitory signals to the thalamus, blocking unwanted motor output. On the contrary, the GPe allows the thalamus to stimulate the motor cortex by inhibiting the stimulatory subthalamic nucleus (STN), which synapses to the GPi [43]. Together, the direct and indirect pathways allow for fine regulation of motor control by dopamine.

SNpc DA neurons receive input from the somatosensory/motor cortex and the subthalamic nucleus, whereas VTA DA cells receive inputs from the lateral hypothalamus. Moreover, dopaminergic neurons in both areas integrate information from the ventral and dorsal striatum, sharing interconnected input [44].

### 2.3. Morphology of Dopaminergic Neurons

The dopaminergic neurons that constitute the VTA and SNpc are different in morphology (size and shape) and the number of connections that a single neuron makes with the striatum. In adult mice, the neurons localized around the midline and in the VTA; in particular, the medioventral portion have been shown to be smaller and rounder, compared to the larger and more angular and elongated cells that comprise the SNpc [45]. Moreover, in rats, a single DA neuron in the SNpc forms 100,000–250,000 connections (in the dorsal striatum), whereas a single DA neuron in the VTA forms only 12,000–30,000 connections in the ventral striatum/prefrontal cortex [46,47]. Considering that the human striatum is about 300 times larger than that of rats, but the number of DA neurons of SNpc only increases by 32 times from rats to humans, this means that each SNpc DA neuron in humans generates a higher number of connections compared to those in rats [46,47]. This assumption is a simplification since the ratio is not a sufficient indicator by which to estimate the number of connections required to cover that region. It is reasonable to speculate that due to the complex arborization and larger synaptic size, SNpc dopaminergic neurons could be more active, with greater metabolic demand, compared to VTA DA cells [47]. Another characteristic of SNpc DA neurons, described by Pacelli in 2015, is that SNpc DA neurons show higher basal respiration compared to VTA DA neurons. SNpc DA neurons seem to work at maximal capacity under baseline conditions, whereas VTA neurons can increase their activity when required, seemingly making VTA neurons less vulnerable to changes in oxygen consumption. Moreover, SNpc DA neurons have a higher axonal mitochondria density and elevated ROS production and an increased vulnerability to different neurotoxins (MPP^+^ (1-methyl-4-phenylpyridinium), rotenone, and H_2_O_2_) [48].

### 2.4. Electrophysiology of DA Neurons

In vitro studies of the electrophysiology of DA neurons in the SNpc have revealed a pacemaker activity property. When neurons are removed from afferent input, the electrophysiology measured in rat brain slices displays pacemaker activity characterized by spontaneous, relatively slow (2–10 Hz), and highly regular firing activity with action potentials of long duration (>2 ms). Another characteristic of the electrophysiology of SNpc DA neurons is a voltage- and time-dependent depolarization component which develops during membrane hyperpolarization and is supported by hyperpolarization-activated current (Ih) and autoinhibition through the high-affinity DA D2-like receptor family [49,50]. Moreover, all SNpc DA neurons display constant Ih to maintain pacemaker activity, a slow APs, prominent after hyperpolarization, the capacity of a regular spontaneous AP activity, and a voltage-dependent inward rectification [51,52,53]. In rodents, a posterior–medial subpopulation has been identified in the VTA that displays little or no Ih currents. Moreover, a subpopulation of VTA DA neurons lacks D2-receptor-mediated autoinhibition, a property present in all SNpc DA neurons. The diversity of the electrophysiological properties of VTA DA cells seems to correlate with their localization and projection [54,55,56].

The firing cycle of dopaminergic neurons requires the coordination of a myriad of ion channels. Adult SNpc DA neurons mostly depend on L-type Cav1.3 Ca^2+^ channels for their pacemaker activity, whereas VTA DA cells mainly rely on sodium channels [57,58]. A very elegant study in 2007 demonstrated that the blocking of Cav1.3 Ca^2+^ channels in adult neurons using isradipine has a neuroprotective effect, both in vitro and in vivo, because it allows neurons to “rejuvenate”, reverting back to a Ca^2+^-independent mechanism to generate their pacemaker activity [59]. Another significant difference between these two classes of DA neurons is that SNpc neurons have modest calcium ion buffering ability, while VTA DA cells express higher levels of the calcium buffering protein calbindin-D28K (not found in SNpc DA neurons), allowing them to sequester calcium without consuming ATP [60].

It is important to mention that there is functional diversity in intrinsic electrophysiological properties, not only between DA neurons in the VTA and SNpc but also in the DA neurons of the same area (for more information about this refer to J. Roeper, 2015 [61]).

### 2.5. Differentiation and Development of A9 and A10 Dopamine Neurons

The midbrain DA (mDA) neurons are generated in the floor plate region of the mesencephalon during embryonic development in a highly coordinated process that requires the timed expression of specific transcription factors (TFs). In rodents, rostral SNpc DA neurons are born around E11.5, while caudal VTA neurons begin around E12.5 [62]. This temporal regulation of the diversity in mDA neurons is conserved between rodents and primates and seems to be controlled by sonic hedgehog (SHH) and fibroblast growth factor 8 (FGF8), which exhibit a biphasic pattern of expression in the floor plate, generating a cartesian axis that defines positional information necessary for mDA neuron induction [63,64]. As mentioned above, the SNpc and VTA have a heterogeneous composition; this is highlighted when we need to discuss development and differentiation, where the time of expression and the transcription factors that are expressed are not constant inside all the VTA and all the SNpc. To explain this complex concept in an easy way, we can consider the time-dependent SHH expression. An early SHH expression is associated with the expression of G protein-coupled receptor kinase 2 (GIRK2) in the rostral SN, while a later SHH expression is associated with generation neurons positive to calbindin in the caudomedial VTA [65]. These two populations represent the endpoint of this temporal wave of expression, and they are characterized to the most-different properties in the adult brain [55]. This heterogeneity inside the VTA and the SNpc will be discussed further below.

Up to embryonic day 13.5 (E13.5) of mouse embryogenesis, SN and VTA midbrain DA precursor cells appear indistinguishable, although these two populations of DA neurons have different markers already at the neuronal progenitor cell stage, such as the transcription factor SOX6 (or SRY-box 2), expressed mainly in SN and orthodenticle homeobox 2 (OTX2), and zinc finger protein 503 (NOLZ1), expressed in VTA precursor [66].

The first transcription factor involved in this differentiation process is OTX2, which is expressed around E7.5. Together with gastrulation brain homeobox 2 (GBX2), which is expressed in the hindbrain, OTX2 helps to define the midbrain–hindbrain boundary [67]. The expression of OTX2 induces Wnt1 expression, while GBX2 induces FGF8 expression. The distribution of FGF8 in the floorplate induces the midbrain fate by stimulating the expression of SHH [68,69,70].

Based on conditional knockout (ko) and overexpression studies in mice, it has been suggested that OTX2 is important for the generation of VTA DA neurons [71,72]. In adult mice, expression analysis has revealed that OTX2 is approximately six-fold enriched in the VTA compared to SNpc DA neurons. This enrichment in VTA neurons seems to be confirmed in humans as well. Neurons positive for OTX2 are often also positive for calbindin [73,74,75,76]. Furthermore, repression of OTX2 in the VTA leads to increased vulnerability to MPTP, a compound used to induce symptoms of Parkinson’s disease (PD). This demonstrates a potential neuroprotective role of OTX2 in VTA neurons in mice, but one that does not seem to be maintained in humans [74,75].

Another crucial transcription factor involved in mDA neuron development is orphan nuclear receptor related 1 (NURR1), which is expressed at E10.5 in mice. NURR1 is the most significant differentially expressed gene, with a high negative correlation with brain aging and Parkinson’s disease (PD) [77]. In ko NURR1 mice, the final differentiation of dopaminergic precursor cells into neurons is inhibited, and SNpc and VTA dopaminergic neurons are not generated in the midbrain. These mouse models develop until gestation but die soon after birth, underscoring the importance of NURR1 in development [78]. Furthermore, the expression of NURR1 needs to be continuously maintained throughout adulthood to preserve the dopaminergic neurons of the SN. In contrast, VTA dopaminergic neurons can survive without NURR1 in adult life [79]. Shortly after NURR1 expression, the expression of tyrosine hydroxylase (TH) is induced, around E11.5. This occurs in parallel with pituitary homeobox 3 (PITX3), whose expression is also regulated by NURR1. TH and PITX3 are both expressed in mDA neurons of SN and VTA. PITX3 is mainly expressed in DA neurons of the ventral SNpc and approximately in 50% of VTA DA [80,81]. In PITX3-deficient aphakia mouse mutants, midbrain dopaminergic neurons appear normal until E12.5. However, this model shows a selective loss of dopaminergic neurons only in the substantia nigra pars compacta (SNpc), while the ventral tegmental area (VTA) is much less affected. Specifically, in this model, the VTA initially develops normally, and degeneration starts to appear in adulthood at 3 months of age. The neuronal loss observed in the VTA, however, is less severe than in the SNpc [82].

Two other genes with extremely different expression patterns in the VTA and SNpc, as mentioned before, are NOLZ1 and SOX6, which appear around E11.5. SOX6 is selectively expressed in neurons belonging to the SNpc. In adults, its expression overlaps with the expression of GIRK2, glycosylated DAT (dopamine transporter), and partially with ALDH1A1 (aldehyde dehydrogenase) genes. This expression pattern is confirmed in rodents and in humans [66]. On the other hand, NOLZ1 is expressed selectively in the VTA at the embryonic stage and disappears at postnatal day 7 [83].

It is also relevant to mention Forkhead box protein A 1 and 2 (FOXA 1/2) and Engrailed-1 and -2 (En1/2). FOXA 1/2 are important for development and differentiation [84,85], and they continue to be expressed in adulthood [86]. The conditional ablation of these two genes in adulthood causes a decrease in ALDH1A1 expression and a complete loss in DAT, leading to a decrease in the number of DA neurons of the SNpc in aged mice [87]. En1/2 proteins are crucial for the maintenance of adult DA neurons, even if the precise mechanism is not completely elucidated. Together with PITX3, genetic variants coding for EN1 are proposed as a risk factor for PD [88,89].

**Figure 2:**
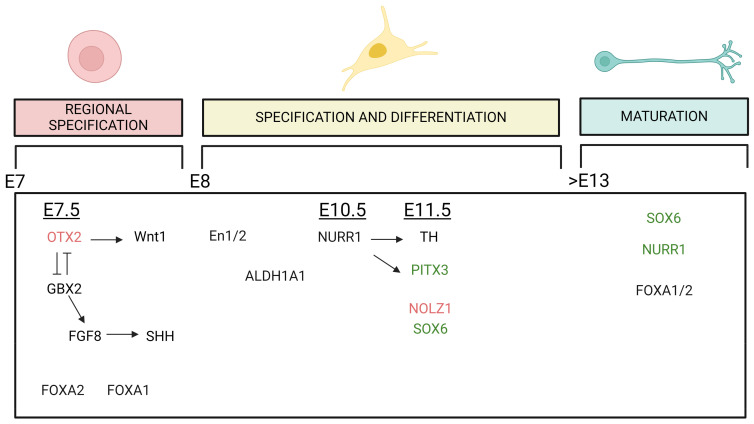
Graphic summary of the TFs involved in the development of mDA neurons that seem to have an impact in the differentiation of DA neurons of the VTA and SNpc. The expression of TFs plays roles in midbrain regional specification, specification and differentiation and maturation of the mDA phenotype, and it is exhibited across various embryonic (E) stages. The temporal line extends from E day 7 to beyond day 13. Arrows indicate a stimulatory effect, while perpendicular lines indicate an inhibitory effect of the TFs. TFs in black do not have a specific regional distribution pattern, while those written in red (VTA) or green (SNpc) colors indicate TFs whose regulation has a higher impact on the development or maintenance of VTA or SNpc DA neurons, respectively. Created with BioRender.

A multitude of studies confirm that the differential expression of these transcription factors and the timing of their expression are characteristic elements, although none of these identified TFs is sufficient, histologically, to differentiate between the VTA and the SNpc accurately (Figure 2). However, the complete characterization of all the processes involved, and more importantly, how to use this knowledge to better understand the higher vulnerability of SNpc DA neurons compared to those in VTA, remains elusive (for more detail on the developmental events of dopaminergic neurons, refer to Garritsen et al., 2023 [26]). Nevertheless, this understanding is undoubtedly a step forward in being able to recapitulate the development of these two areas during differentiation and formation. In the future, this knowledge could potentially provide insights into PD pathology and pave the way for the development of novel therapeutic strategies.

### 2.6. Subpopulation of DA Neurons in the SNpc and VTA

As mentioned before, DA neurons in the SNpc and VTA not only exhibit differences between the two areas but also show variability inside each area. Thanks to single-cell profiling techniques, which allow the analysis of the characteristic expression of individual cells, it has been discovered that the SNpc and VTA have different subtypes of neurons with intrinsic characteristics. In 2014, the first study using this technique identified 96 genes that were differentially expressed in VTA and SNpc neurons in mice. Based on this expression pattern, the authors were able to identify four classes of DA neurons in the VTA and two in the SNpc [90]. Subsequent studies have conducted similar analyses, resulting in different annotations of cell clusters, both in rodents and in humans [91,92,93,94]. Kamath et al., for example, in 2022, developed a new approach to selectively enrich DA neurons from the postmortem human SNpc and analyzed them using single-nucleus RNA sequencing (snRNA-seq). Through this new approach, they identified a subpopulation in the SNpc marked by the AGTR1 gene that seems to have a higher susceptibility to neurodegeneration in PD. This work, along with many others, underscores the importance of identifying specific markers of vulnerability [95]. However, future works are needed to understand which pathways these markers are involved in. This review will not delve deeply into this cluster classification, but it is worth mentioning that thanks to the introduction of single-cell analysis and the evolution of more-sensitive techniques, it has been discovered that the heterogeneity within DA neurons is more profound than previously speculated [95].

### 2.7. Disorders Associated with the SNpc and VTA

**Figure 3:**
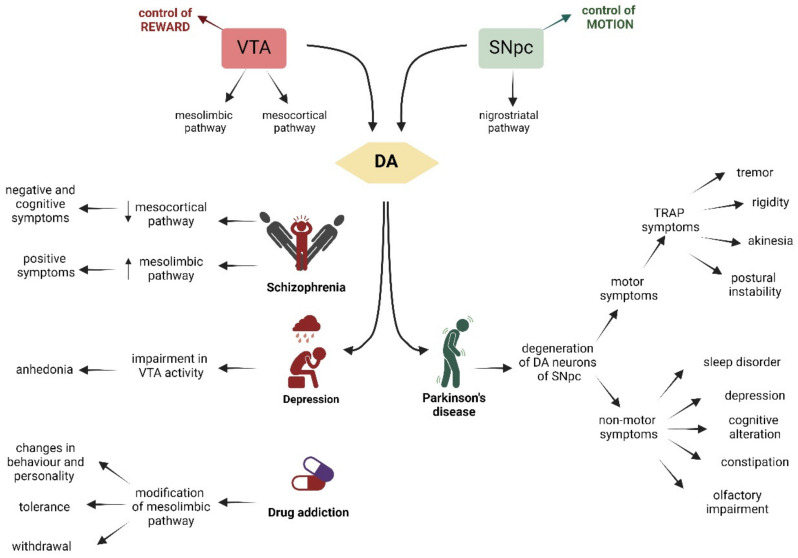
Schematic representation of the VTA on the left (in red), whose main role is the control of reward, and on the right (in green), of the SNpc, which controls motion. The VTA and the SNpc are linked to the production of DA (yellow). VTA is connected through the mesocortic pathway to the cortex and through the mesolimbic pathway to the NAc and striatum. Dysfunctions of these pathways are associated with schizophrenia, depression, and drug addiction. The SNpc controls the voluntary movement and projects via the nigrostriatal pathway to the dorsal striatum. Degeneration of DA neurons of the SNpc is associated with Parkinson’s disease. The characteristic symptoms of each pathology are indicated by arrows. Created with BioRender.

The DA neurons in the SNpc and VTA have different outputs, input projections, electrophysiological activity, and development, but they share the important ability to produce and release dopamine. This makes it very peculiar that these two areas are associated with different disorders. Even though these pathologies initially appear very different, they share certain characteristics that make treatment even more complicated. VTA DA neurons are correlated with reward response, decision-making, incentive salience, stimulus salience, and aversion. Impairment in this area is associated with schizophrenia, major depression, and drug addiction. On the other hand, DA neurons in the SNpc primarily control voluntary movement, and the degeneration of these neurons is the primary cause of Parkinson’s disease (PD) (Figure 3). Here, we will discuss the mechanisms underlying these diseases.

#### 2.7.1. Schizophrenia

Schizophrenia (SZ) is a severe mental illness characterized by the presence of negative symptoms (asocial behavior and impaired motivation) and cognitive symptoms (disorganized thinking and dysfunctions of memory), as well as positive symptoms (delusion and hallucination) [96,97,98]. This pathology affects 1% of the word population and is more frequent in men than in women. The onset occurs in late adolescence or in later life (after age 50) [97,99,100,101,102], although it is thought that it has its origins in the pathological development of the nervous system during the early childhood years [22]. Schizophrenia, in contrast to PD discussed below, is not due to the death of a particular set of mDA neurons; instead, the disease is related to the disruption of the system that provides afferent control of these neurons. In the brain of SZ patients, the activity of the mesocortical pathway that projects from the VTA is decreased, causing a cortical hypodopaminergic state, which is associated with negative and cognitive symptoms. Moreover, this reduction in prefrontal cortex (PFC) function increases the dopaminergic projection onto the limbic area, leading to a hyperdopaminergic state correlated with the positive symptoms of SZ [103,104,105]. The impairment in the dopaminergic system, together with the serotonergic system, seems to be the primary cause of SZ. Supporting this hypothesis is the fact that second-generation (atypical) antipsychotics, which are serotonin–dopamine antagonists, affect both negative, cognitive, and positive symptoms. In contrast, first-generation (typical) antipsychotics, which share antidopaminergic activity, lack an effect on negative symptoms [106].

#### 2.7.2. Drug Addiction

Drug addiction, like SZ, is associated with the VTA system. Almost all the drugs of abuse, despite having different protein targets and mechanisms of action, induce the release of dopamine in the NAc from the VTA, causing a sensation of pleasure [107,108,109].

The physiological function of the VTA and NAc link is to facilitate the motivation required for survival, such as food and sex [110]. By artificially causing a build-up of dopamine in the NAc, the drug of abuse generates an artificial reward effect [111]. After chronic exposure and repetitive release of dopamine, a permanent modification of mesolimbic pathways, genes transcription, epigenetics, neuronal pathways, and neurotrophic mechanisms is induced, leading to a situation where normal physiological pleasures no longer provide the same level of satisfaction [112,113,114].

It is known that only a portion of people, depending on the type of drug, will develop drug addiction after several administrations. This vulnerability is probably linked to genetic and environmental factors [115,116]. Without a mechanistic understanding of drug addiction and why some individuals are more vulnerable than others, developing new therapeutic strategies and providing help for these patients will be very difficult.

#### 2.7.3. Major Depression

Major depression is a syndrome with a spectrum of behavioral symptoms. Two of the key symptoms are the loss of motivation/interest and the reduction in the perception of pleasure (anhedonia), and there is strong scientific evidence that both of these seminal functions are, in part, subserved by VTA dopaminergic neurons [117,118,119,120]. A rat model of depression, induced by exposure to unpredictable chronic mild stress, has been shown to result in an approximate reduction of 50% of VTA neuron activity, primarily in the medial VTA, which is the region correlated with reward function [121]. This model is associated with increased immobility in the swim test and a reduction in sucrose consumption, both of which are commonly used behavioral tests for depression in rodents [122,123,124]. It is easy to speculate that subpopulations of VTA DA neurons could be selectively activated or inhibited in response to depressive stimuli. This could explain the limited efficacy of current antidepressive drugs that target all DA neurons of the VTA equally [125,126,127,128,129,130].

#### 2.7.4. Parkinson’s Disease

Parkinson’s disease (PD) is the second-most-common neurodegenerative disorder, characterized by the degeneration of the dopaminergic neurons in the SNpc. Specifically, analysis of postmortem PD human brains demonstrates a selective loss of DA neurons in the SNpc, with a survival rate of only 10% [131]. There is a higher vulnerability of DA neurons localized in ventral lateral part of the SNpc [132]. In contrast, VTA DA neurons have a higher survival rate (around 60%), with the median and rostral part being even less affected (only 5–25% of the DA cells are lost) [27,133,134]. The death of dopaminergic neurons and the imbalance in the dopaminergic activity in the striatum causes a variety of complex motor symptoms that can be grouped under the acronym “TRAP”: resting tremor, rigidity, akinesia (or bradykinesia), and postural instability. In addition, patients with PD develop flexed posture, motor blocks, and catalepsy, the impairment of movement initiation [135,136]. Before the onset of motor symptoms, patients with PD present non-motor symptoms, which include sleep disorder, depression, and cognitive alteration, as well as constipation and olfactory impairment [137,138,139]. The pathological hallmark of Parkinson’s disease (PD) is the presence of Lewy bodies, which are composed of α-synuclein, neurofilaments, and molecular chaperones [140]. However, there are documented cases of Parkinson’s disease pathology without the presence of Lewy bodies [141]. The average age of PD onset is 55 years, and the risk for developing PD increases five-fold after the age of 70 [142]. Although the majority of patients with PD are sporadic, it is now well understood that some genetic factors contribute to the pathogenesis of PD. Genetic variation contributes approximately 25% to the overall risk of developing PD [143,144,145]. Moreover, there are a number of mutations in single genes that are sufficient to the cause disease, i.e., the familiar forms of PD. Examples of such genes include *SNCA*, encoding α-synuclein [146]; *PARK2*, encoding parkin [147]; *PINK1*, encoding for PTEN-induced putative kinase 1 [148]; *LRRK2*, encoding for leucine-rich repeat kinase 2 [149]; *DJ-1* [150]; and others. The discovery of these monogenic forms of PD helped us to understand more about the mechanisms underlining the susceptibly of the SNpc and to develop models with which to study the pathology and a possible treatment.

Many hypotheses have been proposed to explain the vulnerability of this subpopulation of neurons and the mechanisms underlying neuronal degeneration. These include oxidative stress, abnormal dopamine metabolism, mitochondrial dysfunction, disruption of proteasome or autophagy process, alpha-synuclein aggregation, neuroinflammation, and many others. The reality is that all these processes are impaired and partially linked to each other. However, the exact trigger that causes the neuronal death remains unknown. For a more in-depth discussion of all possible mechanisms implicated in the vulnerability of DA neurons in the SNpc for PD, please refer to Xu Dong-Chen et al. [151].

#### 2.7.5. Treatment Side Effects

Even though it was proposed 60 years ago, the most effective drug available to date to treat PD is the dopamine precursor, L-DOPA, discovered in 1961 by Birkmayer and Hornykiewicza [152]. Treatment with L-DOPA has several problems, however, including it only being a palliative intervention for symptoms; it does not halt the degeneration of DA neurons. Furthermore, it is associated with side effects, including the induction of schizophrenic-like symptoms, such as paranoia and hallucinations, in a significant number of patients after 5 years of treatment [153]. The mechanism of action of this side effect is not yet known. Moreover, the administration of L-DOPA has been linked to the evocation of catatonia, a condition characterized by a trance-like state, posturing, or maintenance of a physical position for a prolonged period of time [154]. Interestingly, according to the *Diagnostic and Statistical Manual of Mental Disorders* (DSM-IV), schizophrenia is categorized into five subtypes, one of which is catatonic schizophrenia [155]. A similar problem exists with antipsychotic drugs used to treat schizophrenia. Antipsychotic agents, especially first-generation agents (FGAs), have an incidence of extrapyramidal side-effects (EPS), such as pseudoparkinsonism, ranging from 15% to 36%. This adverse effect occurs more in older patients, who are already at higher risk for PD [156,157]. The principal mechanism of action of the antipsychotic-induced EPS is the blocking of dopaminergic D2-like receptor family signaling in the nigrostriatal pathway [158]. The fact that the drugs used to treat these two different diseases do not distinguish between classes of dopamine neurons means that desired effects in one condition can become the adverse effects in another.

**Figure 4:**
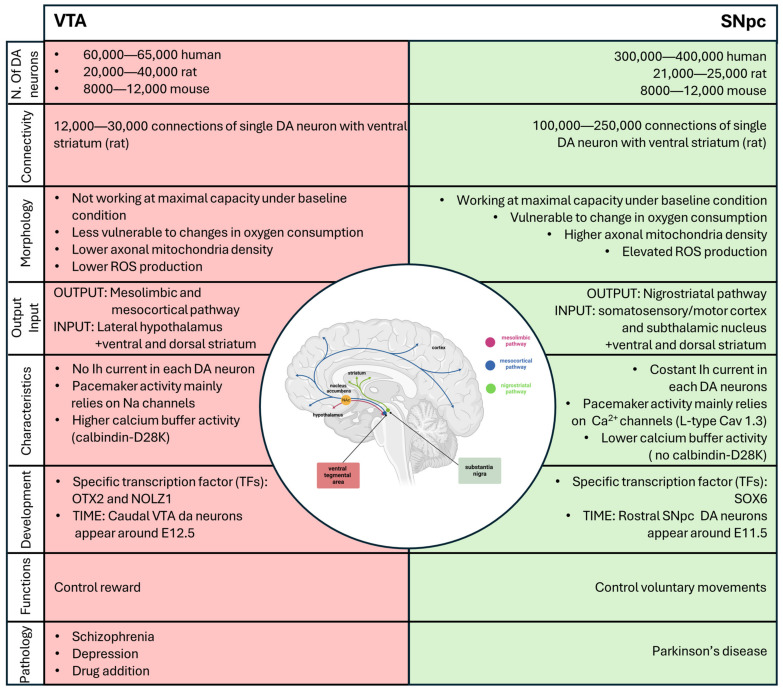
Schematic summary of all the differences between DA neurons of the VTA (in red) and the SNpc (in green). Created with BioRender.

Until recently, it remained puzzling as to how the single dopaminergic neurotransmitter system could be responsible for such disparate symptoms and apparently unrelated diseases. Now, we know that to bridge the current gap in our knowledge, it is crucial to study the functional properties of different DA neuron populations within the intact brain. This will allow us to investigate different brain areas and their mechanisms of action in association with their interconnected network. Understanding the differences between these two areas (Figure 4) is essential for developing treatments that do not inadvertently affect the other structure, thereby minimizing collateral effects. Moreover, comprehension of how these networks function together will provide valuable insights into the mechanisms underlying various neurological and psychiatric disorders.

To take a step forward, we must advance the methods used in this field. In the following section, we will present the current techniques that have enabled us to acquire our existing knowledge, discuss their advantages and limitations, and highlight what is essential for improvement in order to make significant advances in this field.

## 3. Models and Techniques Used to Study Similarity and Differences between the SNpc and the VTA

### 3.1. In Vivo and In Vitro Models to Study the SNpc and VTA

**Figure 5:**
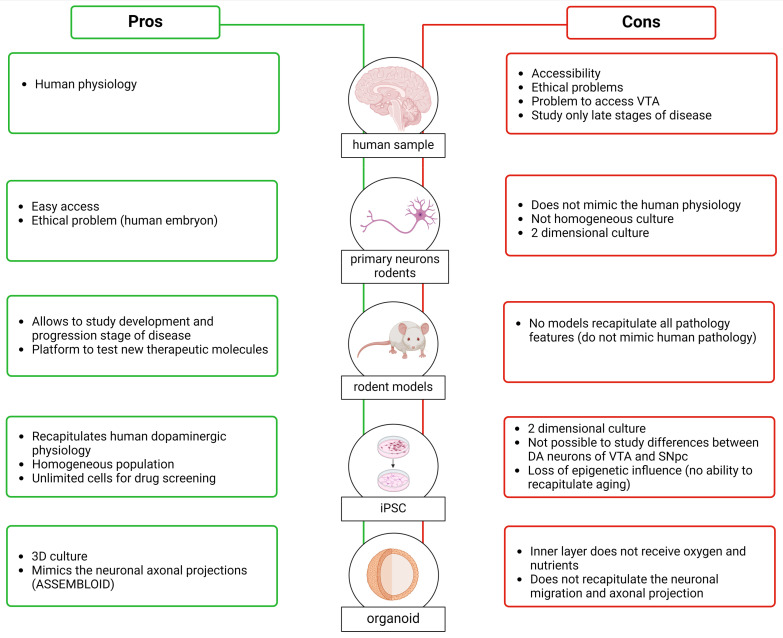
Pros (in green) and cons (in red) of the samples used to investigate the characteristics of DA neurons and study the differences between the DA neurons of the VTA and SNpc. Created with BioRender.

In this section of the review, we will focus on the techniques and methodologies used to study the SNpc and VTA. Our current understanding of these brain regions comes from various sources, including human brain studies, animal models, and in vitro models. Each of these approaches provides unique insights and has distinct advantages and limitations. Additionally, each of these models can be used to produce different types of data, such as transcriptomic and proteomic information (Figure 5). Together, these diverse pieces of information, achieved via several models, contribute to a comprehensive understanding of DA neurons in the SNpc and VTA.

#### 3.1.1. Human Brain

Studying the human brain to understand the SNpc and VTA and their links to neurodegenerative diseases and psychiatric disorders affecting millions of people worldwide seems the most obvious approach. Analysis of human brains, however, is associated with difficult accessibility to material and ethical concerns [159]. The information we have from human brains primarily focuses on analyzing the differences between SNpc DA neurons in patients with PD versus controls rather than understanding the differences between the SNpc and the VTA.

Another important point is that many human brain banks have problems providing tissue samples containing VTA DA neurons. This is because the normal practice is to divide donated brains into two hemispheres, and since the VTA is localized along the midline, the tissue in this region is often damaged. Analyses of human brains are, of course, conducted postmortem, and sometimes, the long postmortem delay can induce protein degradation [160,161]. Regarding experiments aimed at analyzing postmortem PD brains, the information obtained is related only to the late stage of the disease, without providing any information about phenotypic differences and changes in DA neurons at early stages, which are the key stages to understanding how the pathology develops and progresses. Moving beyond these issues, thanks to the histological analysis conducted on the brains of patients in PD, it was possible to find an increase in Cav1.3 subtype expression in the cerebral cortex during the early stages of the disease, before the appearance of symptoms. This supports the idea that impairments in Ca^2+^ homeostasis are an early feature of the disease [162].

#### 3.1.2. Animal Models of PD, SZ, and Depression

To overcome limitations associated with relying purely on studying the human brain, research over the decades has utilized rodent brains to obtain information with the aim of extrapolating it to humans. Moreover, researchers have developed animal models to study various pathologies, such as PD, schizophrenia, and depression. These models aim to understand the mechanisms underlying pathology progression and provide a platform to test new therapeutic molecules. The use of animal models gives us probable information about differences between DA neurons of the SNpc and VTA. The generation of animal models can be achieved through pharmacological/neurotoxic approaches, such as the administration of 6-hydroxydopamine (6-OHDA) [163,164], paraquat (N,N′-dimethyl-4-4-4′-bypiridinium) (PQ) [165,166], rotenone [167], and MPTP for PD, as well as amphetamine [168] and 6-OHDA for schizophrenia [169], alongside genetic approaches. The discovery of familiar forms of PD caused by mutations in specific genes has led to the development of several genetic animal models through the overexpression or downregulation of these genes or their mutated forms. These include models involving α-synuclein (α-syn) [170], LRRK2 [171,172,173], PINK1 [174,175], parkin [176], and DJ-1 [177]. Moreover, genome-wide association studies have reported various schizophrenia risk loci that have been used to create animal models [178], such as Disrupted-in-Schizophrenia 1 (DISC1) [179], dysbindin-1 [180], neurotrophic factor neuregulin 1 (NRG1) and its receptor, epidermal growth factor receptor 4 (ErbB4) [181]. For studying depression, animal models have been developed by applying stress during the developmental period or adulthood or through genetic modification or by selectively breeding for “depressive-like” phenotypes [182]. Like PD and schizophrenia, depression has a strong genetic component, as observed by a meta-analysis conducted in 2019, which identified 269 genes associated with depression [183]. For more information about the models developed to study PD, schizophrenia, and depression, refer to Khan et al. (2023), Białoń et al. (2022), and Planchez et al. (2019), respectively [184,185,186].

Considering that PD, schizophrenia, and depression are multifactorial diseases likely correlated with a particular genetic background and chronic exposure to toxins or stress, it is important to note that the controlled environment of animal models is very different from human ones. Nevertheless, the time course of evolution in animal models is much shorter, for practical reasons, compared to the degeneration time observed in humans; while the development of murine dopaminergic system can be achieved in days, the TH human dopaminergic system develops over months, opening a crucial and vulnerable time window for potential perturbations. Moreover, unfortunately, no mammalian model has been established that can recapitulate all the pathological, etiological, and behavioral features present in human patients [187,188,189]: models remain poor but essential approximations of human pathology. The advantage of these models, in contrast to human brain analysis, is that they can provide information about the early stages and the progression of the disease in the SNpc and VTA. It is crucial to acknowledge their limitations, but the use of animal models provides a significant advantage in studying key steps in the progression of pathologies and in examining differences between the two areas. Additionally, as mentioned earlier, they help to overcome the limitation posed by the availability of human brain samples.

#### 3.1.3. In Vitro Models

In parallel to the use of animal models, in the last decade, a lot of progress has been made in in vitro models. Early studies in this area used primary dopaminergic cultures established from embryonic murine ventral midbrain. Once removed from the mouse brain, the cells are cultured in a specific medium, and here, the isolated neurons rapidly differentiate and form synapses in vitro. This model replicates very well the morphology and physiology of neuronal cells [190]. Moreover, in terms of proliferation rates, these are very similar to human neurons, and they can be considered terminally differentiated neurons [191]. As in animal models, however, they cannot replicate human physiology, anatomy, gene expression regulation, and drug metabolism [192]. Moreover, this culture system is not a homogeneous system since primary dopaminergic cultures usually contain a large number of glia cells and other types of neurons, with TH-positive cells comprising around 5% of the total population [190]. This heterogeneity could affect the reproducibility of the experiments, which is the primary reason why the use of a primary neurons culture is not optimal for the large-scale screening of compounds for therapeutic use. Moreover, the performance of genetic manipulation—for instance, removing or overexpressing a gene associated with PD—is quite challenging due to the low efficiency of transfection or transduction in primary neurons [190,193]. This model is a two-dimensional cellular culture that does not mimic complex aspects of in vivo physiology (primary neurons can also be used to produce organoids, i.e., 3D structures that are presented in the following section).

To overcome the problem that mouse neuronal cells do not recapitulate human physiology, and the ethically complicated use of embryonic human tissue that requires the destruction of embryo, Takahashi et al. and Yu et al., in 2007, described a method for preparing human induced pluripotent stem cells (iPSC) [194,195]. To produce these cells, human somatic cells, such as fibroblasts isolated from a skin biopsy, are reprogrammed using four genes (OCT4, Sox2, Klf4, and c-Myc). Once iPSCs are generated, they can be directed to specific phenotypes, including neural identities, through the timed application of instructive signals. Following neural induction, typically via dual-SMAD inhibition, ventral midbrain progenitors can be specified by the induction of ventral and caudal identity and then matured to differentiated dopamine neurons [194,195]. The neurons obtained from iPSC demonstrate appropriate TH and DAT expression, and they also show spontaneous synaptic activity [196]. Using this approach, it is possible to obtain a PD in vitro model, starting from biopsies from patients that carry a disease-associated mutation. These techniques provide a source of unlimited cells that can be used for drug screening or cell-based therapy for PD [197,198,199,200]. A limitation associated with reprogramming is the loss of epigenetic influence and the ability to recapitulate age related effects. Recent studies, however, have started to identify molecular approaches for aging iPSC-derived neurons [201,202].

Cultures of iPSC-derived dopamine neurons can be grown in either 2D monolayer or 3D organoid format. Their arrangement into a 3D organ-like structure provides the opportunity to explore human brain development in vitro [203,204,205]. Sozzi et al. (2022) developed a method of generating ventral midbrain (VM) organoids [206]. They observed that after long-term culture, these structures exhibit features typical of DA neurons, such as neuromelanin pigmentation. The model described by Sozzi et al. can mimic the architectural organization and functional properties of DA neurons, including post-mitotic molecular features and mature electrophysiological profiles. Organoids also present challenges; for example, central areas in larger structures may not have sufficient access to nutrients in culture media. While a possible solution is the incorporation inside of the organoids of microfluid channels [207,208], this type of organoid system does not accurately recapitulate neuronal migration and axonal projection patterns observed in the brain. To overcome these problems, Reumann (2023) developed an assembloid, which is the combination of ventral midbrain, striatum, and cortical organoids from iPSCs (MISCOs) [209]. This structure displays the interaction between different brain regions via the formation of long projections from the ventral midbrain into the striatum and cortex, which takes into consideration the connectivity of midbrain dopaminergic neurons with their target regions. Reumann (2023) also studied the effect of cocaine administration, showing that artificial elevation of dopamine induces a morphological and transcriptional exchange that persists after stopping the administration. This observation indicated long-term neuronal circuit change of the dopaminergic system during development through dopamine overstimulation [209]. The assembloid overcame the problem of “simple” organoids, generating the possibility of studying the interconnection areas; however, in this case, the deeper layers received less oxygen and nutrition, as observed in organoids. Moreover, MISCOs do not fully recapitulate the in vivo architecture—or, at least, we do not completely know if the axonal innervation we see in this structure follows the same cellular specificity that was seen in vivo. What is clear is that these complex structures seem to be a good substitution or complementation for animal models, which are limited by their inherent physiological differences to the human brain [209].

All the models presented in this section can be used to obtain and extract different information, such as gene and protein expression profiling. These data can be used to identify pathways that are expressed in sensitive neurons and that can be correlated with the vulnerability of the SNpc or pathways expressed in the VTA that can have protective roles, providing an indirect indication of the differences between the VTA and the SNpc.

### 3.2. Techniques to Phenotype SNpc and VTA DA Neurons

#### 3.2.1. Sample Preparation

The first element to take into consideration for an experiment is the sample preparation, which needs to be chosen carefully, based on the scope of the project. One powerful technique for isolating heterogeneous cell populations is laser-capture microdissection (LCM) [210]. LCM offers numerous advantages, including the precise separation of small cell populations or even single cells from a heterogeneous mix, as well as the ability to preserve tissue morphology during dissection. However, LCM also has disadvantages, such as its high cost and often poor RNA yield and quality [211]. Alternatively, fluorescence-activated cell sorting (FACS) has been used to isolate DA neurons, but this method can introduce stress that potentially alters the molecular signature of the cells [212]. A more effective approach may involve the use of Dat bacTRAP mice, which allows for the cell-type-specific isolation of translated mRNAs from DA neurons in vivo without prior fixation, staining, dissociation, or sorting [213]. The preparation of the samples is the first step to a further analysis of gene or protein expression.

#### 3.2.2. Transcriptomics

A popular approach to identifying differences between the DA neurons of the SNpc and VTA is the comparison of gene profiling of these two areas. Comparative expression profiling of human samples is, for the reasons described in the previous chapter, extremely rare, while there are several studies that use rodents to identify the gene expression of the SNpc and VTA. Three different groups in 2004 and 2005 published the use of microarray in rodents (mouse and rat) to analyze gene expression in the SNpc and VTA (Table 1). All three groups noticed the high similarity in gene expression between these two areas (with only 1–3% of the detected genes showing different expression). In each study, the authors tried to link the genes with a particular function; for example, Greene et al. identified that the genes upregulated specifically in VTA encoded proteins involved in neuronal plasticity and survival [73,214,215]. The microarray technique used in these studies helps us to investigate the genes that could be upregulated or downregulated in different areas in physiological states. The primary problem of this methodology is the lack of sensitivity to detect genes which are expressed at low levels. Moreover, it has a limited dynamic range of detection, with a low signal-to-noise ratio and an upper limit to detection of transcripts, beyond which the signal becomes saturated. More recent approaches to RNA sequencing have, however, made in-roads in finding a good alternative to overcome these problems.

The analysis of the three studies described above relies on the idea that the SNpc and VTA are composed of homogeneous molecule populations. The evolution of RNA sequencing in combination with single-cell analysis allowed us to investigate gene expression with high sensibility and take into consideration the heterogenicity of the samples. Indeed, Poulin and colleagues, in 2014, demonstrated the existence of subtypes of DA neurons using single-cell RNA analysis, with a characteristic expression profile. The advantage of single-cell RNA sequencing is profiling of the SNpc at single-cell resolution both in rodents and in humans [91,93,94]. Although this technique gives us a significant advantage in terms of our knowledge of the DA neurons in the SNpc and VTA, it is also associated with some problems. Cell dissociation is a critical step that affects the molecular profiles because it can induce the expression of stress genes [216]. To overcome this problem, a good replacement is single-nuclei RNA sequencing (snRNA-seq). This technique can also operate at a very low level of mRNA in a single brain tissue nucleus [217]. The snRNA-seq was used also by Kamath and colleagues in 2022 to analyze DA neurons in the SNpc in the human post-mortem [95], where a new protocol was proposed to selectively enrich the DA neurons from the post-mortem human SNpc.

Transcriptomic analysis gives us important information but, alone, it might not be sufficient to define closely related DA neuron subtypes. This limit could be overcome in combination with a multi-omics approach. Spatial transcriptomics allows us to better understand the spatial architecture within the brain structure. This technology gives positional information of the gene expression data, allowing for the generation of an illustrated map of the cell subtype inside the tissue. An example of the application of this technique can be found in the study conducted by Aguila et al. [218]. The authors used the spatial transcriptomics method LCM-seq, which combines laser capture microdissection (LCM) with Smart-seq2 [219] RNA sequencing to analyze single DA neurons of the SNpc and VTA from 18 human post-mortem brains. They identified seven differentially expressed genes (DEGs) in the SNpc and twenty-one DEGs in the VTA (Table 2) [218,220]. The DEGs identified in the SNpc were previously identified in two independent human microarray datasets which only analyzed SNpc neurons [221,222]. Moreover, in the same work, the authors showed that using too small a sample size could be a problem and a possible reason for the variability observed among the previous studies [73,214,215,220]. Very importantly, the authors showed that some of the identified DEGs were also differently expressed in patients with PD, suggesting that these markers can be used to study the VTA and SNpc in both healthy and in patients with PD.

In addition to human and rodent tissue samples, transcriptomic analysis can also be conducted in in vitro models. A project from 2022 analyzed the gene expression of a patient with PD after generating human pluripotent stem cells (hiPSCs) carrying the ILE368ASN mutation within the PINK1 gene and control using single-cell RNA sequencing (RNAseq) [223]. In this work, the authors analyzed four different time points of differentiation in order to identify the pathways altered in the early stage of the pathology. The analysis of the genes links to ubiquitination, mitochondrial function, protein processing, RNA metabolism, and vesicular transport.

#### 3.2.3. Proteomics

Most molecular profiling studies of DA neurons in the SNpc and VTA have focused on mRNA. While this approach has significantly enhanced our understanding of gene expression in these neurons, it cannot provide all the necessary information. mRNA levels do not always correlate with protein levels, and transcriptomics cannot establish the localization of encoded proteins within specific subcellular compartments. For these reasons, it is also important to obtain information about protein expression. Proteomics can be performed in a range of biofluids, tissues, or cellular models. Similar to transcriptomic studies, these studies often compare protein expression between control and patients with PD using post-mortem samples or brain samples obtained from rodent PD models.

For instance, a study conducted in 2004 by Basso and colleagues compared the proteins in the SN of patients with PD with those in controls. They identified 44 proteins in these samples using 2-DE and MALDI-TOF analysis, and 9 of these proteins showed changes in expression between the PD and control groups [224]. Another study, conducted just two years later, utilized a proteomics technique called shotgun proteomic multidimensional protein identification technology (MudPIT) to quantify mitochondrial proteins in patients with PD. They identified 119 proteins with different expression levels between PD and control samples. Subsequently, they used a cellular model of PD, consistently treated with rotenone, to confirm some of the differentially expressed proteins identified in the mass spectrometry experiment [225]. Two years later, Werner and colleagues used MALDI-TOF to identify proteins up- or downregulated in the SNpc of patients with idiopathic PD compared to control. They identified 37 proteins, of which 16 were differentially expressed [226] (Table 3).

The Zhang group conducted another proteomic study using the iTRAQ labeling technique to analyze differential protein expression in four groups: the VTA area in controls and in patients with PD, and the SNpc area in controls and in patients with PD [227]. They then combined this last study with the previous one and compared the obtained results with those of Basso et al. They confirmed that 75% of the 44 proteins identified by Basso and colleagues were also identified in their studies, validating the reliability of their results. Additionally, they compared their studies conducted in humans with another proteomic work that analyzed differential protein expression in the SNpc of control and MPTP mouse models [228]. They observed a partial overlap of the proteins identified in mice with those identified in humans. However, they also found that most of the proteins identified in the human brain were not found in the mouse model, demonstrating again that analyses in rodents cannot perfectly recapitulate aspects of the human brain.

Proteomic analyses of brain tissue typically involve a heterogeneous mixture of cell populations, including glia, astrocytes, oligodendrocytes, and others, with only a small fraction represented by neurons. Craft et al. (2013) proposed a method by which to isolate a single population by fractionating brain tissue [229]. This procedure reduces the heterogeneity of the analyzed cells but also decreases the number of proteins available for the experiment, which can limit the reliability of the results. Additionally, the fractionation process is not perfect, and the final samples may contain impurities. Another approach to overcoming this problem is using in vitro models where the analyzed population of cells is homogenic. In a recent work, Novak used PINK1-ILE368ASN neurons, a PD model, to conduct proteomic analysis. Specifically, the authors first analyzed the transcriptome in this in vitro model and then conducted proteomic analysis to confirm whether the observations at the transcript level corresponded to functional deficits [223]. The combination of these two techniques is a good example of how separate methodologies, each providing different types of information, can be used to support, confirm, and enhance each other’s results. This approach, as demonstrated in the paper by Novak and colleagues, shows how transcriptomic and proteomic analyses can be integrated to provide a more comprehensive understanding of the biological processes and functional deficits in a PD model. Another way to enhance the information obtained from proteomic technology is to combine it with more-innovative procedures. One such example is the analysis by mass spectrometry (MS) of synapse-enriched fractions (synaptosomes) conducted by van Oostrum et al. [230], which took advantage of the fluorescence-activated synaptosome sorting (FASS) procedure developed by Biesemann and improved it for a system-wide analysis [231]. Using Cre-inducible knock-in mice and FASS coupled with MS, the diversity of the synaptic proteome was investigated across genetically defined synapse types and brain areas. Focusing on an in-depth analysis of the synaptic proteome of striatal dopaminergic terminals, the study identified 267 significantly enriched proteins at dopaminergic terminals. By comparing these results with 14 other synaptic proteomes, it was proposed that the absence of Oxr1, a protein that protects against oxidative stress, could contribute to the high vulnerability observed in PD.

In summary, different models used to study the SNpc and VTA, as well as investigations into why the DA neurons of the SNpc are more vulnerable to PD, indicate that each model has advantages and disadvantages. Moreover, the techniques discussed above (although they are not exhaustively listed) provide useful—albeit incomplete—information. The take-home message is that the models and techniques should be combined, and it is essential to corroborate the results obtained with one technique using other methods as well. Additionally, future studies should consider the knowledge obtained from past research. For example, Aguila et al. showed that the minimal sample size required to identify the markers differentially expressed between SNpc and VTA dopamine neurons reliably was eight subjects [218].

## 4. Allen Brain Atlas

Another tool available to researchers is the Allen Brain Atlas database, which serves as a vital resource by providing comprehensive molecular brain maps (https://atlas.brain-map.org/, accessed on 28 August 2024). This database provides human and mouse maps, as well as maps of the developing mouse brain and a small part of developing human brain. An example of the use of the information available in the Allen Brain Atlas is reported below.

In the Allen Brain Atlas database, within the human brain section, we used the differential search function to identify genes which are more highly expressed in the SNpc compared to the VTA in six different donors. Then, the reverse analysis was also performed (more highly expressed in VTA compared to the SNpc). Due to the large number of genes identified, only the 2000 genes with higher fold change for each donor were selected for comparison, with each gene being associated with a *p*-value.

The 2000 genes from each donor were compared to identify genes that are conserved across all donors (Supplementary Table S1). In the next step, these two lists of genes higher—those expressed in the SNpc compared to the VTA and those higher expressed in the VTA compared to the SNpc—were crossed first with a list of genes found downregulated in the SNpc of patients with PD compared to controls in five studies found in the literature (Supplementary Table S2), and then with the genes found upregulated (Supplementary Table S3) in the same studies (Bossers et al., 2009; Yang et al., 2022; Verma et al., 2023; Zhou et al., 2023; and Huang et al., 2024) [232,233,234,235,236]. To note, some of these studies used a common microarray dataset. The rationale behind the comparison of differentially expressed genes in PD was to identify genes that are differentially abundant or detected only in cells that are more vulnerable or to find genes that may have a protective role.

The comparisons made in this example are summarized in the Supplementary Table S4.

The first analysis compared genes that were more highly expressed in the SNpc compared to the VTA with the genes that were lower expressed in patients with PD compared to the controls (Supplementary Figure S1 and Supplementary Table S5). This analysis showed that many genes that are highly expressed in the SNpc compared in the VTA are also downregulated in the SNpc of patients with PD.

In the next step, we compared, using Venn diagrams, all the genes identified previously (Supplementary Figure S2). All the genes that were found to be higher expressed in the SNpc compared to the VTA and that are also downregulated in the SNpc of patients with PD in at least two studies are reported in Supplementary Table S6. Three genes were found in all five studies (AGTR1, ALDH1A1, and GBE1), and six were found in four studies (OLFM3, PCDH8, DOK6, RET, KLHL1, and FGF13).

The same procedure as explained previously was followed to identify the common genes between those with higher expression in the VTA compared to the SNpc (Supplementary Table S1) and those with lower expression in patients with PD compared to controls (Supplementary Table S2). The comparisons show only one gene, FOXA2, which is constant in at least two lists (Supplementary Figure S3 and Supplementary Table S7).

No genes were found in common when we cross-referenced the genes with higher expression in the SNpc compared to the VTA and the genes with higher expression in the VTA compared to the SNpc (Supplementary Table S1) with the genes found to be upregulated in patients with PD compared to controls (Supplementary Table S3).

**Figure 6:**
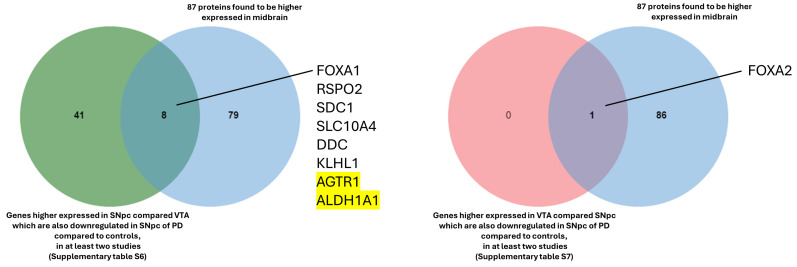
Proteins found enriched in the midbrain and reported in the Allen Brain Atlas. The two highlighted genes are found in all five studies (Bossers et al., 2009 [232]; Yang et al., 2022 [233]; Verma et al., 2023 [234]; Zhou et al., 2023 [235]; and Huang et al., 2024 [236]).

For functional insights, the genes identified through the comparisons (Supplementary Tables S6 and S7) were compared with the 87 proteins reported in the Allen Brain Atlas that are enriched in the midbrain (https://www.proteinatlas.org/humanproteome/brain/midbrain, accessed on 28 August 2024), revealing the following common genes and proteins: FOXA1, SDC1, RPSO2, DDC, SLC10A4, KLHL1, AGTR1, and ALDH1A1 in the SNpc; and FOXA2 in the VTA (Figure 6).

Reassuringly, the two genes identified in all five studies (AGTR1 and ALDH1A1) are biologically significant, emphasizing the relevance of this comparison. AGTR1 was found to be spatially confined to the ventral tier of the SNpc, the region more susceptible to loss in PD [95]. Another study demonstrated that the inhibition of ALDH1A1 precedes the loss of DA neurons in the SNpc and may render those neurons more susceptible to degeneration [237].

## 5. Conclusions

Even though our knowledge about the similarities and differences between DA neurons of the VTA and SNpc has increased, it seems insufficient to generate a treatment for one of the diseases associated with these areas without indirectly affecting the other and creating collateral effects, as evidenced by reports for schizophrenia treatment that induce Parkinson’s-like symptoms [156,157]. One of the crucial points of this review is the underlining of what is known about the phenotypic differences between DA neurons of the SNpc and VTA, but we also point out the importance of studying the correlation of these two areas as opposed to only focusing our scientific efforts on the comparison between patient and control. The discovery of pathways or characteristics that make one nucleus more vulnerable or more resistant, for example, to PD can lead to further analyses in PD models, while always bearing in mind that it is not only the VTA and the SNpc that have differences; there is heterogeneity within each area [90].

The second part of this review analyzes the models and the techniques used to study the differences between the DA neurons of the VTA and SNpc. When analyzing the literature that characterizes the DA neurons of the SNpc and VTA, the results often show differences and incongruences. These discrepancies can be attributed to the use of different species in the studies, indicating variations in brain structure and organization, even within primates, as well as in mice and rats. While rodent models provide valuable information about the development of the different DA neurons, their brain organization and functionality differ from humans. In addition to the aforementioned factors, the differences in sample preparation methods contribute to inconsistencies across studies.

While it is well established that SNpc and VTA neurons govern distinct functions (movement and reward/motivation, respectively [23,238]) the distinction between these areas is not sharply defined given their proximity and interconnection. Single-cell analyses have highlighted that even within these regions, DA neurons exhibit varying characteristics. For instance, within the SNpc, neurons in the ventral tier are more vulnerable than those in the dorsal tier and the VTA [132]. Recent single-cell transcriptomic studies have identified genes that are differentially regulated in more susceptible areas, providing markers that can be used to isolate and further analyze these specific cells [95].

In this context, the Allen Brain Atlas database (https://portal.brain-map.org/, accessed on 28 August 2024) is already a valuable tool for researchers, but it could be even more advantageous if the maps were extended beyond mice and humans to include other species, different developmental stages, and diseased brains. Employing high-throughput techniques such as single-cell RNA sequencing and Slide-seq would facilitate these expansions. Additionally, integrating proteomics alongside transcriptomics, which are already available, would allow for the mapping of proteins as well as mRNAs, offering a more-detailed understanding of the molecular landscape.

The goal of future research should be to leverage this knowledge to distinguish between DA neuron subtypes and develop more selective treatments. One example is the use of markers identified through single-cell transcriptomics, combined with the bacTRAP technique [213]. Designing bacTRAP constructs that use promoters of genes identified as specific markers (e.g., AGTR1) [95], researchers can create transgenic mice where ribosomal tagging occurs specifically in the targeted DA neuron subpopulations. This allows for the selective isolation and analysis of translating mRNAs from these vulnerable neurons, integrating both transcriptional and translational data for a comprehensive molecular understanding. Furthermore, the discovery of new drugs requires the capability to conduct high-throughput drug screening. The relevance of these screenings depends on the extent to which in vitro models replicate human physiology. Therefore, substantial improvements in in vitro models are essential to investigate new targets and drugs effectively. These models need to better mimic the connections, heterogeneity, and organization of the human brain to make the screening processes more relevant and predictive of clinical outcomes.

## Figures and Tables

**Table 1:** Comparison between three studies that use microarray in rodents (mouse and rat) to analyze gene expression in the SNpc and VTA. The genes reported are identified in at least two of the three studies. The “X” indicates the study in which the gene was identified. The underlined genes were found in all the three studies. Grimm et al., 2004 [214] and Greene et al. (2005) [215] used mouse samples, while Chung et al. (2005) [73] used rat samples.

	Grimm et al., 2004 [214]	Chung et al., 2005 [73]	Greene et al., 2005 [215]
**Enriched in SN**	Rat samples	Mouse samples	Rat samples
Fgf1		X	X
Gad1		X	X
Grin2c	X	X	X
Igf1		X	X
Prkcd	X		X
Slc25a5		X	X
Sncg	X	X	X
Sox6	X	X	
Spp1		X	X
**Enriched in VTA**			
Adcyap1		X	X
Adra1b	X		X
Calb1		X	X
Egr1	X	X	
Grp		X	X
Lpl	X	X	X
Marchs	X		X
Olfm1	X		X
Plod2	X		X
Slc17a6	X	X	
Spint2	X		X
Tacr3		X	X

**Table 2:** Summary of the common human genes identified by Nichterwitz et al., 2016 [220] and Aguila et al. (2021) [218], which are detected only in the VTA and SNpc samples, respectively. Nichterwitz analyzed single DA neurons of the SNpc and VTA from three human post-mortem brains.

Analysis of Single Human DA Neurons of SNpc and VTA					
VTA				SNpc	
	Cadm1 Fxyd6	Zcchc12 Serpine2	Crym Cdh13		Gsg1l Atp2a3
	Peg3	Kcnip4	En2		Vat1
	Cacna2d3	Pcsk2	Osbpl3		Rgs16
	Loc728392	Arhgap26	Necab1		Zfhx2
	Ptchd1	Timp2	Stc1		Cbln1
	Gng4	Ly6h	Peg10		Slit1

(female, age 47, 50 and 52 years old, cause of death: breast carcinoma, bronchocarci-noma and leiomyosarcoma, respectively), while Aguila used 18 human post-mortem brains from 18 males with ages between 45–102 years old (for the case of death refer to Supplementary Table 2 of the study [216]).

**Table 3:** Summary of results of the proteins found to be differentially expressed in the SNpc neurons of patients with PD compared to controls in two different studies.

Different Protein Expression in Snpc of PD Patients Compared Controls			
Basso et al., 2004 [224]		Werner et al., 2008 [226]	
Upregulated in PD	Downregulated in PD	Upregulated in PD	Downregulated in PD
Peroxiredoxin II	L neurofilament	Ferritin H	V-type ATPase A1
Mitochondrial complex III	M neurofilament	Glutathione-S-transferase (GST) M3	
ATP synthase D chain		GST P1	
Complexin I		GST O1	
Profilin		SH3-binding glutamic acid -rich like protein (SH3BGRL)	
L-type calcium channel d-subunit		Glial fibrillary acidic protein (GFAP)	
Fatty-acid binding protein		Glial maturation factor beta (GMFB)	
		Galectin-1	
		Sorcin A	
		S-adenosyl homocysteine (SAHcy) hydrolase 1 (L-DOPA methylation)	
		Aldehyde dehydrogenase A1 (ADH1A1)	
		Cellular retinol-binding protein 1 (CRBP1)	
		Annexin V	
		Beta-tubulin cofactor A	
		Coactosin-like protein 1	

## Data Availability

Not applicable.

## Supplementary Materials

The following supporting information can be downloaded at https://www.mdpi.com/article/10.3390/biology13090690/s1: Supplementary Figures S1–S3 and Supplementary Tables S1–S7. Supplementary Table S1: List of the genes higher expressed in SNpc compared to VTA and genes higher expressed in VTA compared to SNpc. Genes obtained matched the first 2000 genes higher expressed (higher fold-change) of the 6 donors. Supplementary Table S2: Summary table of all the genes found downregulated in SNpc in PD patients compared to controls in five different studies (Bossers et al., 2009 [232]; Yang et al., 2022 [233]; Verma et al., 2023 [234]; Zhou et al., 2023 [235]; and Huang et al., 2024 [236]). Supplementary Table S3: Summary table all the genes found upregulated in SNpc in PD patients compared to control in five different studies (Bossers et al., 2009 [232]; Yang et al., 2022 [233]; Verma et al., 2023 [234]; Zhou et al., 2023 [235]; and Huang et al., 2024 [236]). Supplementary Table S4: Summary of the comparisons of the study. Supplementary Figure S1: Venn diagrams that visually show the number of common elements found between genes (A, B, C, D and E) higher expressed in SNpc compared VTA and Genes downregulated in SNpc in PD patients compared to controls of five different studies found in literature (Bossers et al., 2009 [232]; Yang et al., 2022 [233]; Verma et al., 2023 [234]; Zhou et al., 2023 [235]; and Huang et al., 2024 [236]). Supplementary Table S5: Summary table of common elements found using Venn diagrams (Supplementary Figure S1) between genes higher expressed in SNpc compared to VTA and genes downregulated in SNpc in PD patients compared to controls of five different studies found in literature (Bossers et al., 2009 [232]; Yang et al., 2022 [233]; Verma et al., 2023 [234]; Zhou et al., 2023 [235]; and Huang et al., 2024 [236]). Supplementary Figure S2: Venn diagram between the A, B, C, D and E groups (Supplementary Table S5: Common elements found between genes higher expressed in SNpc compared VTA and genes downregulated in SNpc in PD patients compared to controls of five different studies find in literature (Bossers et al., 2009 [232]; Yang et al., 2022 [233]; Verma et al., 2023 [234]; Zhou et al., 2023 [235]; and Huang et al., 2024 [236]). Supplementary Table S6: Summary table of Venn diagram of Supplementary Figure S2. In the table the genes higher expressed in SNpc compared VTA which are also downregulated in SNpc of PD compared to controls are reported. The colons indicate in how many studies the genes were found. Only the genes higher expressed in SNpc compared to VTA and found downregulated in SNpc in PD patients compared to control in all five study (AGTR1, ALDH1A1, GBE1). Supplementary Figure S3: Venn diagram that visually show the number of common elements found between genes higher expressed in VTA compared SNpc and genes downregulated in SNpc in PD patients compared to controls in five different studies found in literature (Bossers et al., 2009 [232]; Yang et al., 2022 [233]; Verma et al., 2023 [234]; Zhou et al., 2023 [235]; and Huang et al., 2024 [236]). Supplementary Table S7: Summary table of common elements found using Venn diagrams (Supplementary Figure S3) between genes higher expressed in VTA compared SNpc and genes downregulated in SNpc in PD patients compared to controls of five different studies found in literature (Bossers et al., 2009 [232]; Yang et al., 2022 [233]; Verma et al., 2023 [234]; Zhou et al., 2023 [235]; and Huang et al., 2024 [236]).
