# Supplementary material for: State of the Art in Sub-Phenotyping Midbrain Dopamine Neurons
Source: Biology (Basel). 2024 Sep 3;13(9):690. doi: 10.3390/biology13090690 (PMC11428604; doi:10.3390/biology13090690)
Supplement: Supplementary file 1 [file biology-13-00690-s001.zip › biology-3168194-supplementary.pdf]

Supplementary File

State of the Art in Sub-Phenotyping Midbrain Dopamine Neurons

by **Valentina Basso** [1,], **Máté D Döbrössy** [2,3,4], **Lachlan H Thompson** [5,6], **Deniz Kirik** [7], **Heidi R Fuller** [8,9,*] and **Monte A Gates** [1,*]

1 School of Medicine, Keele University, Staffordshire ST5 5BG, UK; v.basso@keele.ac.uk
2 Laboratory of Stereotaxy and Interventional Neurosciences, Department of Stereotactic and
Functional Neurosurgery, Medical Center, University of Freiburg, 79106 Freiburg im Breisgau, Germany;
mate.dobrossy@uniklinik-freiburg.de
3 Department of Stereotactic and Functional Neurosurgery, Medical Center, University of Freiburg,
79106 Freiburg im Breisgau, Germany
4 Faculty of Biology, University of Freiburg, 79104 Freiburg im Breisgau, Germany
5 Charles Perkins Centre, Faculty of Medicine and Health, School of Medical Sciences,
The University of Sydney, Sydney, NSW,2006 Australia;
lachlan.thompson@sydney.edu.au
6 Aligning Science Across Parkinson's (ASAP) Collaborative Research Network,
Chevy Chase, MD 20815, USA
7 Brain Repair and Imaging in Neural Systems (B.R.A.I.N.S) Unit, Department of Experimental
Medical Science, Lund University, BMC D11, 22184 Lund, Sweden;
deniz.kirik@med.lu.se
8 School of Pharmacy and Bioengineering, Keele University, Staffordshire ST5 5BG, UK
9 Wolfson Centre for Inherited Neuromuscular Disease, TORCH Building, RJAH
Orthopaedic Hospital, Oswestry SY10 7AG, UK
* Correspondence: h.r.fuller@keele.ac.uk (H.R.F.); m.a.gates@keele.ac.uk (M.A.G.);
Tel.: +44-1691-404693 (H.R.F.); +44-1782-733875 (M.A.G.)

Genes higher expressed in SNpc compared to VTA				Genes higher expressed in VTA compared to SNpc		
LMOD3	A_24_P511187	FAM19A3	VAT1	PENK	S1PR5	FGFR2
PYGL	SV2C	LMO3	DOK6	SCGN	NEUROD2	COL21A1
COL12A1	ZAR1	PCDH8	SYT17	CHODL	TRIM59	GAD1
CYP2S1	A_32_P133106	KCNQ2	A_32_P17525	GLRA2	A_23_P69368	OXR1
RSPO2	RESP18	PRKAR2B	ABHD2	DIRAS3	RASGRP1	SOX14
AGTR1	A_23_P206120	DDC	A_32_P35969	SLC6A4	F13A1	RYR2
SMPX	A_23_P417646	GBE1	CACNB3	CHRNA3	NPTX1	IGFBP3
SLITRK4	CYP27C1	SNX25	GPR98	CRH	CNTNAP4	BTG3
CELSR1	ANK1	GSG1L	CDK14	CALB2	CHRM2	GRIK1
F2RL2	RGAG1	OSBPL10	DENND2A	SLC17A6	A_24_P935437	CCNE2
CBLN1	GPR161	C16orf93	LRRC3B	KIT	NGB	PHTF2
RDH12	CUX2	CHRNA4	FAM135B	BAIAP3	A_24_P548966	SLC30A3
NPNT	A_32_P497742	ROBO2	A_24_P624263	A_32_P183652	NKX6-1	A_32_P115840
APOC1	PKIB	A_32_P207481	NCAPG	KCNQ5	KCNAB1	SCN1A
EBF3	PID1	A_24_P722155	LOC100144602	SST	UGT8	GRIA4
SLC10A4	KLHL1	CAB39L	KCNQ3	PTPRT	PCSK6	FOXA2
MATN3	DRD2	TMEM200C	TSPAN6	MYH7	ABCA8	GRM7
CADPS2	A_24_P47211	FGF13	ZNF503	NELL1	GULP1	GATA3
FEZF1	KANK4	MSI2	FST	NXPH2	HIST1H1A	ZEB2
TPBG	CNIH3	A_24_P929202	SNCA	A_32_P3431	NOS1	CACNA1I
GABRA4	RGS4	TUB	CLPTM1L	HTR2C	KCNJ2	DUSP16
A_23_P424269	KLHL13	IZUMO2	SH3BGRL2	COCH	STC1	PCDH11Y
RET	A_32_P130999	LOC441052	CRYGD	RPRM	C21orf91	TOX
ALDH1A1	NTN1	SEMA6D	FAM43B	KCNJ3	GAS7	PNOC
OC90	GFRA1	FLJ30901	PIP5K1B	TMEM200A	TJAP1	LDLR
NTSR1	OLFM3	A_24_P234554	COMTD1	HTR1E	SCD	SSTR2
PROK2	CERS6	A_32_P58407	LOC730091	CNR1	EVI2A	PDYN
HTR1F	GALNTL6	ERC2	SATB1	CNDP1	SORCS1	
ATP2A3	GPR26	PBX1	RARRES1	MYO1E	SYNJ2	
RGS16	ANKRD29	A_24_P410536	RHOF	NPTX2	CBLN4	
FOXA1	LRRC55	LXN	GAL	EGFL6	SEMA3D	
SSTR1	ENPP1	VGLL3		ADRA1D	EHF	
GPRC5A	HPGD	PLEKHA5		OPRM1	GLDN	
KCNJ6	ST3GAL1	MAN1C1		THSD7B	LPPR5	
B3GALT1	ITPR1	POU3F2		DACH1	LGR5	
OXTR	RNF144B	SEZ6L		SORCS3	NECAB2	
RERG	SLC2A13	A_32_P154361		CALCR	SEMA5A	
VAV3	CORO2A	A_24_P707156		SYTL5	C1QL3	
SDC1	MMD	TAB3		GUCY1A3	CHRM3	
A_32_P203688	EFCAB5	FAM71F1		CPNE4	KIAA1239	

Supplementary Table S1: List of the genes higher expressed in SNpc compared to VTA and genes higher expressed in VTA compared to SNpc. Genes obtained matched the first 2000 genes higher expressed (higher fold-change) of the 6 donors.

Genes downregulated in SNpc of PD patients compared to controls (five different studies)																			
Bossers et al, 2009						Yang et al, 2022		Verma et al, 2023							Zhou et al, 2023			Huang et al, 2024	
GBE1	ROBO2	BTBD10	NUDT11	OPTN	SC5DL	SSTR1	HMOX1	MCM10	STAU2	EPHA7	ATP6V1B2	HSPH1	CADPS2	ELOVL3	RALYL	CAMK1G	SUSD1	PDE6H	DLD
KLHL13	C6ORF168	NSF	KIAA0802	ATP6V1H	DNM3	LINC00515	SOWAHA	FEZF1	JPT1	TBC1D9	ACHE	ANK1	HBA2	HBG2	KLHL13	DOK6	CNTN6	CLSTN3	DHRS7B
DYNC1I1	RAP1GDS1	HPRT1	AP3M2	RUFY3	DNAJC12	RBM3	DDX3Y	KCNG3	FABP7	GSG1L	SLC38A2	B3GALT1	CRYGD	SLC10A4	STYK1	KANK4	SLC6A3	VAV3	TBC1D19
PYGL	DKFZP434L 187	PRKAR2B	CLSTN2	CADPS	GPRASP2	MAPK8IP1	RELN	CISD1	APC	DNAJA4	FKBP1B	INSM1	LINC00261	SLC6A3	PCSK1	CUX2	NR4A2	UCHL1	CCNH
CAST1	SNAP91	RAB3GAP1	DSU	INPP4B	GOT1	KDR	TDRD6	PSMA4	TENT4B	BASP1	CHRNA4	SYNGR3	DAPL1	AGTR1	RAB3C	BEND4		ATP6V1H	TAC1
SNCA	PCLO	FGF12	NECAP1	TM2D2	C1ORF33	DNAJB6	CPVL	KCMF1	IRAK2	CPEB3	PCSK1	ANKRD34C	UNC13C	SDC1	SLC8A1	REEP1		CDK5	GCH1
RGS16	ALDH1A1	DYNC1LI1	RPL41	USP14	IMMT	RERG	NR4A2	ATP6V1D	UBE2F	HSPA4L	RIMS1	OLFM3	TH	SLC35D3	SYNGR3	GPR26		KIFAP3	LXN
DENR	MAGI3	PTS	MAP2K4	FRY	RFPL1S	PCDH8	PCSK1	TMEM183A	SPATS2	YWHAG	CHORDC1	CYP27C1	FOXA2		CFAP46	ATP2A3		ATP6VDD1	KLHL1
NETO2	MAGEE1	LOC390616	ATP5B	ITPR1	RIMS2	KLHL1	AKR1C3	MORF4L2	LYSMD2	DAB1	LIPG	GFRA1	RET		SLC2A13	FLJ30901		TUB83	NRXN3
SMPX	DMXL2	PPM1E	RIMS1	FAM70A	ACTR10	AGTR1	GABRA4	MAFG	AKAP12	VSIG10L	CHAC1	C2ORF80	GLRA3		CACNB3	CBLN1		SNX10	PCDH3
TPBG	ST8SIA3	TBC1D9	EEF1E1	TUSC2	COX6B1	TNRC6C-AS1	BCL6	TTC1	CLPTM1L	MAP2K4	PIP5K1B	ATP2A3	GABRA4		CACNA1E	KLHL1		LPPR4	SV2C
RNF31	ATP8A2	KCNS3	C3ORF14	FOXA1	CALN1	SLC10A4	RHOBTB1	ATP6V0B	CDKN2D	CACYBP	GRHL1	PGM2L1	ALDH1A1		SIAH3	GBE1		SCG2	EN1
FST	AP1GBP1	COPS4	KCTD6	DPP6	NDUFV2	CUX2	RNASE2	GFOD2	TMEM70	RIMBP2	RAB3A	TMEM255A	SLC2A13		ISM1	SLC35D3		REEP1	SCN35
AKAP12	MRPS25	ANKRD50	SLC25A4	DHX36	SLC9A6	EBF3	PSPH	ARPC5L	RIPPLY2	RCAN2	FAM43B	CNTN4	RGS4		CORO2A	GPR161		SYNGR3	PEG10
TTMA	LRRC3B	KCNMA1	LRPPRC	PELO	SNX10	TIMM238	LRRC3B	UBFD1	MDH1	SYNJ1	DOK6	LINC01166	NOV		LMOD3	SDC1		SYT1	FGF13
DACH2	KIFAP3	RGS8	ADAM23	ATP5A1	PTPRN2	DDC	VCAM1	DUSP26	HSP90AB1	ACOT7	STMN2	CACNB3	PITX3		FOXA2	SLC18A2		HPRT1	DLK1
TRIM36	UNC13C	PGM2L1	PTPN4	MRPL15	FBXO9	NANOS1	TAC1	ATP1B3	CNIH2	TMEM200C	PRKAR2B	ELAVL2	BEND4		EN1	KCNG3		NECAP1	RET
SLITRK5	DLK1	NAP1L5	MYO5A	C10ORF22	TCP11L1	SLC18A2	C5AR1	ATP6V1G1	ITPR1	CDK14	KHDRBS2	GAS2L3	FAM19A3		CLSTN2	LOC100288 310		RGS4	KCNJ5
SYT1	PCDH18	COL4A3BP	TMEM118	GABBR2	ARG2	PRMT6	ANGPT2	RANBP9	DYNC1I1	DGKH	CADPS	GBE1	EBF3		CATSPERG	C2ORF80		ERC2	TH
EHBP1	HSPA4L	SCG5	LMO3	SLC18A2	MLLT11	ALDH1A1	DOK6	ZC3H15	KPNA2	ZNF503	EFCAB5	RALYL	LPO		SMIM10L2 B	RDH12		TMEM35	SLC6A3
SCN3A	RAB3A	LASS6	FLJ10781	CUGBP2	PLEKHB2	KCNE1L	CTXN3	APOO	B4GALNT1	UCHL1	LINC-PINT	ERC2	GPR26		SLC10A4	CHRNB3		NELL2	SLC18A2
DGKH	VSNL1	UNC13A	LRRN3	GLA	FLJ20701	RSP02	CXCR4	PPTC7	SNRNP48	CMAS	NPM2	MEG8	GALNTL6		CDH8	ALDH1A1		CHGB	DDC
ELOVL4	CMAS	NELL2	ATXN3	AAK1		SLC5A3	SLC35D3	YARS	POPDC3	SNCA	CELF4	RAB3C	ATXN8OS		PLCXD2	LOC441052		AMPH	PCSK1
NR4A2	HN1	PFDN4	DSCR1L1	NAG6		SPA17	FGF13	PTS	TBPL1	GCH1	OSBPL10	RDH12	PCDH8		TPBG	ANK1		CADPS	GBE1
AGTR1	AMPH	BRUNOL4	FAM102B	MOAP1		C2ORF80	KDM5D	CISD2	DNAJC12	VWC2L	CA2	SYT1	SV2C		RET	PCDH8		CNIH3	PID1
RPL41	TIMM8A	PRMT8	SNAP25	RAB3C		TH	CBLN1	PITPNB	ATF4	STX1A	GAP43	NCAPG	FGF13		RSPO2	CPLX2		SMPX	CADPS2
CABYR	SH3BGRL2	UBE2V2	ABCC5	PREPL		HIST1H2BD	FGF12	PITHD1	RBM48	HSP90AA1	HTR2A	ACKR1	DRD2		PGM2L1	SSTR1		RIT2	ALDH1A1
VAV3	SLC6A15	FABP6	PSMD12	STXBP1		GBE1	OLFM3	GARS	CHCHD4	TOX2	KLHL13	VAV3	SMPX		OSBPL10	CYP27C1		NUP93	AGTR1
GLS	REEP1	SUMO3	SLC25A14	RRAGB		C5ORF64	APLNR	ASNS	LINC00888	SMIM10L2 B	CHSY3	GPRC5A	CBLN1		KCNJ6	ROBO2		DCTN3	SLC25A32
SDC2	UBE2T	LOC400451	OSBPL10	GABARAPL 3		UNC13C	COPG2IT1	CEBPG	SNX25	HPRT1	NR4A2	CPLX2	LMX1B		RAB27B	SHANK2		PLD3	MATN3
LOC138046	OCRL	NAPB	RPS6KA3	PCDH8		UHRF1	S100A4	C1ORF52	SNX16	SERGEF	ZNF385D	CKS2	CHRNB3		FST	FGF13		OGDHL	MCAT
NRXN3	CD226	NAP1L2	MAGI1	CRMP1		TMEM255A	FZD7	STRAP	PPA1	PTGS2	TTC39A	RTL9	SLC18A2		DRD2	CHD5		DHDDS	
BCAT1	HPGD	XK	EIF1B	C1ORF71		SDR16C5	OPALIN	RAB39B	TSPAN13	CDKN3	LRRC55	HBA1	FST		RIMBP2	KCNB1		PIN1	
GPRC5A	DOK6	INSM2	BCAS2	GABARAPL 1		ROBO2	LY96	VDAC3	PSMD12	SNCG	SLITRK4	CUX2	KLHL1		B4GALT6	UNC13C		KIAA0319	
HS6ST3	STS-1	SV2B	TMEM35	C16ORF59		CLSTN2	ELAVL2	EEF1E1	KCTD6	NEDD4L	CORO2A	LMOD3	KCNJ6		TTC39C	GFRA1		CITED1	
SCN2A2	MDH1	MAPK9	AXUD1	COX5A		CDH8		CCDC6	BEX5	UBE2T	HPGD	SCUBE1	DDC		EBF3	SV2C		CNTN6	
GABRA4	UBE2F	HSPA12A	SPCS3	PFTK1		GPR26		EHBP1	DENR	TPBG	SSTR1	DLK1	RSPO2		GPRC5A	DCC		CCNA1	
SRPK2	FLJ44635	LRRC49	RAB3GAP1	NMNAT2		MID1IP1		CCT8	RUNDC3A	KCND3	CTXN3	CYP2S1	FOXA1		TUB	DLK1		SSSCA1	
OLFM3	RIT2	PGK1	SNRPN	CPEB3		KCNJ6		PHF23	LINC00599	TMEM35A	LINC01956	EN1	ZAR1		DIRAS2	OLFM3		SLBP	
AKAP12	TRIM37	HRASLS	PRKCB1	RIMS3		RET		MRPL21	TUB	LGI2	CATSPERG	PRMT8	NTSR1		AGTR1	DAPL1		TMEM14A	

Supplementary table S2

Summary table of all the genes found downregulated in SNpc in PD patients compared to controls in five different studies (Bossers et al., 2009 [232]; Yang et al., 2022 [233]; Verma et al., 2023 [234]; Zhou et al., 2023 [235]; and Huang et al., 2024 [236]).

Genes upregulated in SNpc of PD patients compared to controls (five different studies)						
**Bossers et al, 2009**	**Yang et al, 2022**	**Verma et al, 2023**			**Zhou et al, 2023**	**Huang et al, 2024**
SOX2	CD99P1	FABP1	APLN	SPX	HSPA1L	HSPA6
SSH3	RPL3L	MYO1A	VCAM1	HSDL2	HSPA1B	MICB
LTF	PLEK2	POU4F2	AOC3	MICALL1		IRF7
VIM	RAB42	CRLF1	GPRC5C	ADCYAP1R1		DDIT4
PHLPP	DLG3-AS1	NUTM2A	DLGAP1-AS5	SMAD9		BCL6
EDG2	LLNC01101	C1ORF87	LINC01137	DNAJC15		KCNE4
LOC400960	IL13	LINC00702	ATP13A4	C5		IGJ
LRDD	MELK	CCDC170	DNAH17	MARCKSL1		SIPA1
TGIF	PYCRL	CYP4F3	MFAP4	DDX60		AXIN1
PTMA	L EAP2	THNSL2	MRO	GM2A		
RGMA	DNA2	PRLHR	LINGO3	MYH14		
GBP1	PCDHGA8	LINC01549	CMTM5	MCCC2		
PDGFRB	C15ORF37	TMEM30B	ITGA7	MVB12B		
C17ORF27	LOC100288893	FAM131C	LINC00910			
SOX9	LPO	MEGF6	PCDHGA4			
CASP7	LOC100133130	FRMPD2	SLC24A4			
SASH1	NEDD4	IGSF1	IDH2			
CDK2AP1	LOC100130987	ACSBG1	SYN3			
HIP1R	DACH2	EFHC2	CDH23			
PLEKHB1	NTSR1	TOGARAM2	SLC38A3			
ZNF532	PCDHGA1O	LINC02447	ARPIN			
KLK6	ASIC2	PLIN4	HRASLS5			
FBXL7	INSM2	DNAH9	ADA2			
FN1	SNORD114-3	ERBB2	PAMR1			
CTDSP1	LOC400043	C1QTNF4	ZNF710			
LRRC58	RBM11	DNAH5	BTBD16			
MVP	LINC01158	GPD1	SNX22			
MLLT7	CNTN6	SSPN	COL5A3			
CA2	CCT6B	PRODH	NTRK2			
MID1IP1	LOC441052	ZNF843	PCDHGC3			
P2RX7	EN1	CCDC8	CHST6			
S100A13	TTTY15	MMD2	CCDC88C			
FLJ27365	ABCA11P	LINC02177	HCG11			
WWC1	DLK1	SMIM1	MARVELD1			
FYCO1	C21ORF37	ADHFE1	GPR27			
GNAI2	WDR17	SLFN5	DGKG			
ARHGEF2	PIM1	ABCC8	SLC44A2			
DDAH2	KCNE4	TMEM37	PTK2B			
PXN	DAPL1	MORN1	SLC24A1			
DDEFL1	LRRN1	GPR75	RHBDL3			
	DDIT4L	ACTA2	QSOX1			
	SDC1	MYH15	LINC00461			

Supplementary table S3

Summary table all the genes found upregulated in SNpc in PD patients compared to control in five different studies (Bossers et al., 2009 [232]; Yang et al., 2022 [233]; Verma et al., 2023 [234]; Zhou et al., 2023 [235]; and Huang et al., 2024 [236]).

**Genes higher expressed in SNpc compared to VTA**	**Genes downregulated in SNpc in PD patients compared to controls**
	**Genes upregulated in SNpc in PD patients compared to controls**
**Genes higher expressed in VTA compared to SNpc**	**Genes downregulatde in SNpc in PD patients compared to controls**
	**Genes upregulated in SNpc PD patients compared to controls**

Supplementary table S4

Summary of the comparisons of the study.

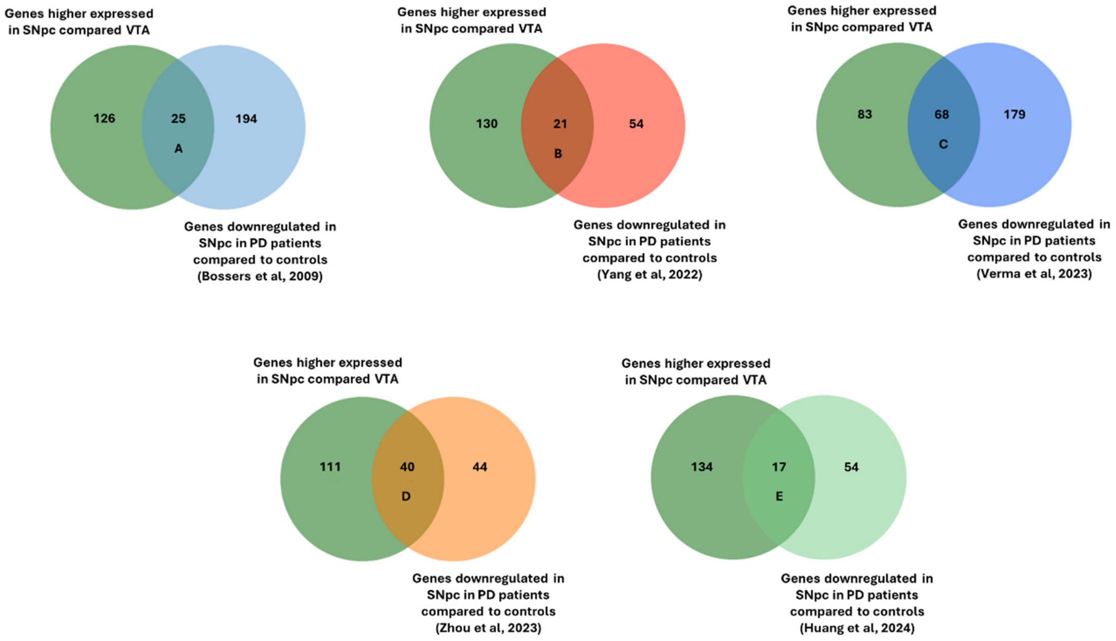

Supplementary Figure S1

Venn diagrams that visually show the number of common elements found between genes (A, B, C, D and E) higher expressed in SNpc compared VTA and Genes downregulated in SNpc in PD patients compared to controls of five different studies found in (Bossers et al., 2009 [232]; Yang et al., 2022 [233]; Verma et al., 2023 [234]; Zhou et al., 2023 [235]; and Huang et al., 2024 [236]).

Common elements found between genes higher expressed in SNpc compared VTA and genes downregulated in SNpc in PD patients compared to controls of five different studies found in literature (Bossers et al, 2009; Yang et al, 2022; Verma et al, 2023; Zhou et al, 2023; Huang et al, 2024)

A	B	C		D		E
PYGL	AGTR1	LMOD3	GFRA1	LMOD3	TUB	AGTR1
AGTR1	CBLN1	CYP2S1	OLFM3	RSPO2	LOC441052	SMPX
SMPX	EBF3	RSPO2	GALNTL6	AGTR1	FLJ30901	MATN3
TPBG	SLC10A4	AGTR1	GPR26	CBLN1	DOK6	CADPS2
GABRA4	GABRA4	SMPX	LRRC55	RDH12	CACNB3	RET
ALDH1A1	RET	SLITRK4	HPGD	EBF3	FST	ALDH1A1
RGS16	ALDH1A1	CBLN1	ITPR1	SLC10A4		VAV3
FOXA1	SSTR1	RDH12	SLC2A13	TPBG		SV2C
GPRC5A	KCNJ6	EBF3	CORO2A	RET		PID1
VAV3	RERG	SLC10A4	EFCAB5	ALDH1A1		KLHL1
KLHL13	CUX2	CADPS2	FAM19A3	ATP2A3		CNIH3
OLFM3	KLHL1	FEZF1	PCDH8	SSTR1		RGS4
HPGD	OLFM3	TPBG	PRKAR2B	GPRC5A		DDC
ITPR1	GPR26	GABRA4	DDC	KCNJ6		GBE1
LMO3	PCDH8	RET	GBE1	SDC1		FGF13
PCDH8	DDC	ALDH1A1	SNX25	SV2C		ERC2
PRKAR2B	GBE1	NTSR1	GSG1L	CYP27C1		LXN
GBE1	ROBO2	ATP2A3	OSBPL10	ANK1		
OSBPL10	FGF13	FOXA1	CHRNA4	GPR161		
ROBO2	DOK6	SSTR1	TMEM200C	CUX2		
DOK6	LRRC3B	GPRC5A	FGF13	KLHL1		
LRRC3B		KCNJ6	TUB	DRD2		
FST		B3GALT1	ERC2	KANK4		
SNCA		VAV3	DOK6	KLHL13		
SH3BGRL2		SDC1	CACNB3	GFRA1		
		SV2C	CDK14	OLFM3		
		ZAR1	NCAPG	GPR26		
		CYP27C1	ZNF503	SLC2A13		
		ANK1	FST	CORO2A		
		CUX2	SNCA	PCDH8		
		KLHL1	CLPTM1L	GBE1		
		DRD2	CRYGD	OSBPL10		
		RGS4	FAM43B	ROBO2		
		KLHL13	PIP5K1B	FGF13		

Supplementary table S5

Summary table of common elements found using Venn diagrams (supplementary Figure S1) between genes higher expressed in SNpc compared to VTA and genes downregulated in SNpc in PD patients compared to controls of five different studies found in literature (Bossers et al., 2009 [232]; Yang et al., 2022 [233]; Verma et al., 2023 [234]; Zhou et al., 2023 [235]; and Huang et al., 2024 [236]).

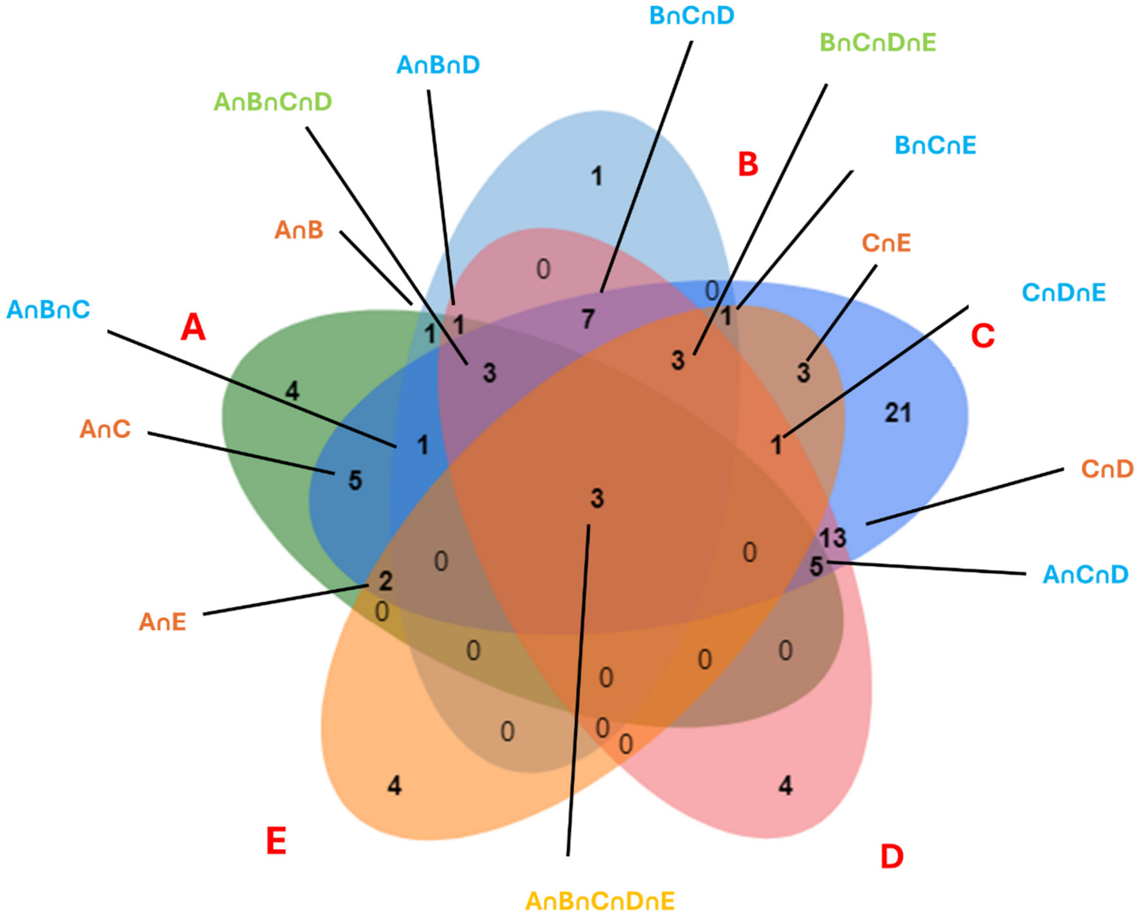

<u>Supplementary Figure S2</u>

Venn diagram between the A, B, C, D and E groups (supplement Table S5: Common elements found between genes higher expressed in SNpc compared VTA and genes downregulated in SNpc in PD patients compared to controls of five different studies find in literature (Bossers et al., 2009 [232]; Yang et al., 2022 [233]; Verma et al., 2023 [234]; Zhou et al., 2023 [235]; and Huang et al., 2024 [236]).

Genes found in 2 studies		Genes found in 3 studies		Genes found in 4 studies		Genes found in 5 studies	
LRRC3B	**A∩B**	GABRA4	**A∩B∩C**	OLFM3<br>PCDH8<br>DOK6	**A∩B∩C∩D**	AGTR1<br>ALDH1A1<br>GBE1	**A∩B∩C∩D∩D∩E**
FOXA1<br>HPGD<br>ITPR1<br>PRKAR2B<br>SNCA	**A∩C**	ROBO2	**A∩B∩D**	RET<br>KLHL1<br>FGF13	**B∩C∩D∩E**		
LMOD3<br>RSPO2<br>RDH12<br>ATP2A3<br>SDC1<br>CYP27C1<br>ANK1<br>DRD2<br>GFRA1<br>SLC2A13<br>CORO2A<br>TUB<br>CACNB3	**C∩D**	TPBG<br>GPRC5A<br>KLHL13<br>OSBPL10<br>FST	**A∩C∩D**				
CADPS2<br>RGS4<br>ERC2	**C∩E**	SMPX<br>VAV3	**A∩C∩E**				
		CBLN1<br>EBF3<br>SLC10A4<br>SSTR1<br>KCNJ6<br>CUX2<br>GPR26	**B∩C∩D**				
		DDC	**B∩C∩E**				
		SV2C	**C∩D∩E**				

Supplementary table S6

Summary table of Venn diagram of supplementary Figure S2. In the table the genes higher expressed in SNpc compared VTA which are also downregulated in SNpc of PD compared to controls are reported. The colons indicate in how many studies the genes were found. Only the genes higher expressed in SNpc compared to VTA and found downregulated in SNpc in PD patients compared to control in all five study (AGTR1, ALDH1A1, GBE1).

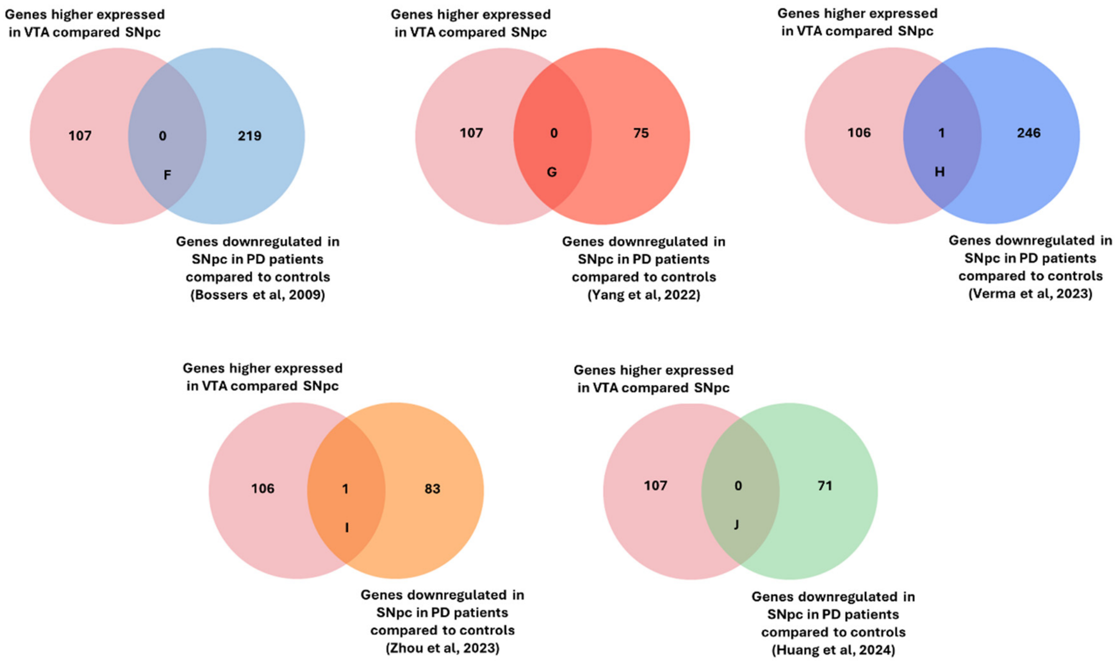

Supplementary Figure S3

Venn diagram that visually show the number of common elements found between genes higher expressed in VTA compared SNpc and genes downregulated in SNpc in PD patients compared to controls in five different studies found in literature (Bossers et al., 2009 [232]; Yang et al., 2022 [233]; Verma et al., 2023 [234]; Zhou et al., 2023 [235]; and Huang et al., 2024 [236]).

**Common elements found between genes higher expressed in VTA compared SNpc and genes downregulated in SNpc in PD patients compared to controls of five different studies find in literature (Bossers et al, 2009; Yang et al, 2022; Verma et al, 2023; Zhou et al, 2023; Huang et al, 2024)**	
**H**	**I**
FOXA2	FOXA2

Supplementary table S7

Summary table of common elements found using Venn diagrams (supplementary Figure S3) between genes higher expressed in VTA compared SNpc and genes downregulated in SNpc in PD patients compared to controls of five different studies found in literature (Bossers et al., 2009 [232]; Yang et al., 2022 [233]; Verma et al., 2023 [234]; Zhou et al., 2023 [235]; and Huang et al., 2024 [236]).
